# Bone sarcoma patient-derived xenografts are faithful and stable preclinical models for molecular and therapeutic investigations

**DOI:** 10.1038/s41598-019-48634-y

**Published:** 2019-08-21

**Authors:** Patrizia Nanni, Lorena Landuzzi, Maria Cristina Manara, Alberto Righi, Giordano Nicoletti, Camilla Cristalli, Michela Pasello, Alessandro Parra, Marianna Carrabotta, Manuela Ferracin, Arianna Palladini, Marianna L. Ianzano, Veronica Giusti, Francesca Ruzzi, Mauro Magnani, Davide Maria Donati, Piero Picci, Pier-Luigi Lollini, Katia Scotlandi

**Affiliations:** 1 Laboratory of Immunology and Biology of Metastasis, Department of Experimental, Diagnostic and Specialty Medicine (DIMES), University of Bologna, Bologna, Italy; 2 Laboratory of Experimental Oncology, IRCCS Istituto Ortopedico Rizzoli, Bologna, Italy; 3 CRS Development of Biomolecular Therapies, Laboratory of Experimental Oncology, IRCCS Istituto Ortopedico Rizzoli, Bologna, Italy; 4 Service of Pathology, IRCCS Istituto Ortopedico Rizzoli, Bologna, Italy; 5 Department of Experimental, Diagnostic and Specialty Medicine (DIMES), University of Bologna, Bologna, Italy; 6 Diatheva srl, Fano, Italy; 7 Third Orthopedic Clinic and Traumatology, IRCCS Istituto Ortopedico Rizzoli, Bologna, Italy; 8 CRS Development of Biomolecular Therapies, Laboratory of Experimental Oncology, IRCCS Istituto Ortopedico Rizzoli, Bologna, Italy

**Keywords:** Bone cancer, Sarcoma

## Abstract

Standard therapy of osteosarcoma (OS) and Ewing sarcoma (EW) rests on cytotoxic regimes, which are largely unsuccessful in advanced patients. Preclinical models are needed to break this impasse. A panel of patient-derived xenografts (PDX) was established by implantation of fresh, surgically resected osteosarcoma (OS) and Ewing sarcoma (EW) in NSG mice. Engraftment was obtained in 22 of 61 OS (36%) and 7 of 29 EW (24%). The success rate in establishing primary cell cultures from OS was lower than the percentage of PDX engraftment in mice, whereas the reverse was observed for EW; the implementation of both *in vivo* and *in vitro* seeding increased the proportion of patients yielding at least one workable model. The establishment of *in vitro* cultures from PDX was highly efficient in both tumor types, reaching 100% for EW. Morphological and immunohistochemical (SATB2, P-glycoprotein 1, CD99, caveolin 1) studies and gene expression profiling showed a remarkable similarity between patient’s tumor and PDX, which was maintained over several passages in mice, whereas cell cultures displayed a lower correlation with human samples. Genes differentially expressed between OS original tumor and PDX mostly belonged to leuykocyte-specific pathways, as human infiltrate is gradually replaced by murine leukocytes during growth in mice. In EW, which contained scant infiltrates, no gene was differentially expressed between the original tumor and the PDX. A novel therapeutic combination of anti-CD99 diabody C7 and irinotecan was tested against two EW PDX; both drugs inhibited PDX growth, the addition of anti-CD99 was beneficial when chemotherapy alone was less effective. The panel of OS and EW PDX faithfully mirrored morphologic and genetic features of bone sarcomas, representing reliable models to test therapeutic approaches.

## Introduction

Osteosarcoma (OS) and Ewing sarcoma (EW), the two most common primary tumors of bone, are high-grade malignant neoplasms with very aggressive behavior and high tendency to form metastasis; they arise frequently in children and remain prominent among teenagers and young adults^1–4^.

Patients are still treated with conventional therapies comprising a combination of high-dose multidrug chemotherapy associated with local control of the tumor by surgery and/or radiotherapy^1,3,5^. As a consequence of this multimodal treatment, patients with localized disease at diagnosis have a 5-year survival rate of nearly 65% for OS and 70% for EW^4,6^. However, patients with disseminated disease at diagnosis and patients who fail first-line treatment have survival rates as low as 30–35%^2,7^. Furthermore, heavy side effects severely compromise the quality of life in these young patients. There is a strong demand from patients, families and oncologists of therapies with improved efficacy and reduced side effects.

Any further improvement in the design of innovative therapeutic approaches requires a better understanding of tumor evolution, development of drug resistance and the testing of new compounds in appropriate experimental models^8–12^. Recently the development of patient-derived xenografts (PDXs) obtained by direct implant of surgically resected tumors in immunodeficient mice, has offered a more accurate and reliable preclinical model of cancer^9,13–18^. We present here a new large panel of OS and EW PDX and primary cell cultures obtained from patients treated at IRCCS Istituto Ortopedico Rizzoli, and we show that PDX faithfully mirror the molecular and cellular phenotype of the original human tumor.

## Methods

### Tissue sampling

Tumor samples were obtained from surgical specimens under sterile conditions. Whenever sample size was deemed to be sufficient, the available material was split into four parts and processed as follows: (1) the tissue to be implanted in immunodeficient mice for the generation of PDX was placed in Iscove’s Modified Dulbecco’s Medium (IMDM) supplemented with 10% Fetal Bovine Serum (FBS) (Euroclone) and antibiotics (penicillin, streptomycin), hereafter referred to as complete medium; (2) tissue for genetic analyses was frozen in liquid nitrogen and stored at −80 °C; (3) tissue for histopathology and immunohistochemistry was fixed in a 10% formalin solution; (4) any remaining tissue was used for *in vitro* cultures (see below).

### Mice and establishment of PDX

Immunodeficient NOD Scid gamma (NSG) mice were bred under sterile conditions in our animal facilities from founders originally obtained from Charles River, Italy. To generate PDXs, a fresh tumor specimen measuring approximately 4 mm^3^ was implanted subcutaneously (s.c.) at the level of trans-scapular brown fat of 5–11-week-old NSG male mice within an average of 1–2 hours following patient’s surgery. Tumor growth was monitored at least twice weekly using calipers until it reached a maximal volume of 2.5 cm^3^, then the mouse was sacrificed by CO_2_ inhalation and cervical dislocation, the tumor was removed, and an accurate necropsy was performed to assess metastatic spread. The tumor was minced with scissors and tumor fragments were implanted in NSG mice; the remaining fragments were immersed in 90% FBS + 10% DMSO for viable storage in liquid nitrogen or used for histopathological and molecular analyses.

### Growth of established PDX in different immunodeficient mice

Established PDXs were also implanted in 5-11-week-old BALB/c Rag2−/−; Il2rg−/− (hereafter referred to as RGKO) mice bred in our animal facilities from founders kindly given to us by Drs. T. Nomura and M. Ito of the Central Institute for Experimental Animals (Kawasaki, Japan)^19^. Equal amounts of PDX fragments, obtained as described above from tumors grown in NSG mice, were implanted in parallel in RGKO mice and in NSG mice. Tumor growth was measured as described above.

### Primary patient-derived or PDX-derived cell lines

Tissue samples obtained from the patient or from a PDX were minced into small pieces and placed in complete medium into 60-mm dishes (Falcon) incubated at 37 °C in a 5% CO_2_ humified atmosphere. When the outgrowth cultures formed a confluent monolayer, the cells were sub-cultured after enzymatic removal with 0.05% trypsin-EDTA and maintained *in vitro* for at least 10 passages, before being processed for *in vitro* studies. Cell lines were authenticated through STR analysis (PowerPlex ESX Fast System, Promega) in comparison to the profile of the original surgical specimen and of the PDX when appropriate, moreover the human origin of *in vitro* cultures was confirmed by PCR analysis with species-specific primers.

### Histopathology and immunohistochemistry

**Figure 1:**
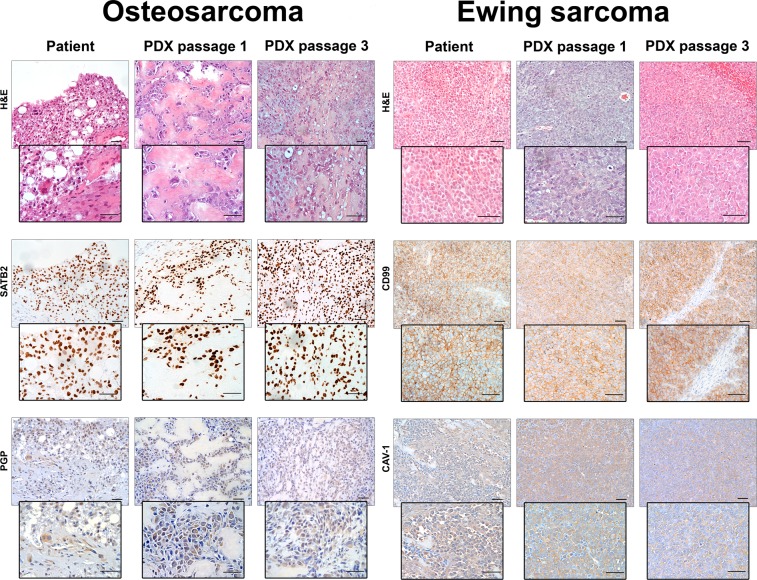
Histologic and immunohistochemical features of patients’ tumors and corresponding PDX at different *in vivo* passages. OS sections were stained with hematoxylin and eosin (H&E) or with antibodies against OS biomarkers SATB2 and PGP. PDXs closely resembles patient’s tumor, including the production of neoplastic bone and the presence of anaplastic cells. SATB2 and PGP expression of PDX mirrored that of patient’s tumor. EW sections were stained with H&E or with antibodies against EW biomarkers CD99 and CAV-1. EW PDXs consist of small round cell sheets, closely packed and without matrix, resembling patient’s tumors. CD99 and CAV-1 expression of PDX mirrored that of patient’s tumor. Bar: 50 μm.

The tissues were fixed in 10% buffered formalin, routinely processed, and embedded in paraffin. Serial, 3-μm-thick, paraffin sections mounted on pre-coated slides were processed according to standardized automated procedures (Ventana Medical Systems, Tucson AZ, USA), and immunostained with the following antibodies: CD99 (Ventana, Mouse Monoclonal antibody O13, pre-diluted), SATB2 (Santa Cruz Biotechnology, Mouse Monoclonal antibody SATBA4B10, 1:200 dilution), RUNX2 (Santa Cruz Biotechnologies, SC-101145 27-K 1:10 dilution), caveolin-1 (BD - Transduction Labs, 610058 1:500 dilution), anti MDR1 P-gp (ABCB1), clone JSB-1 (Monosan - MON9011-1 1:50 diluition), or with buffer alone (negative control). Pretreatment for antigen retrieval was performed at 95 °C with Tris-EDTA, pH 8.00 for 20 minutes. Staining was performed with the UltraView Universal DAB Detection Kit (Ventana Medical Systems, Tucson AZ, USA). Appropriate positive and negative controls were included in each run, furthermore all stained sections included non-tumor mouse cells, such as endothelial cell, myopericytes and fibroblasts which were invariably negative (*see* Fig. 1). For morphological analyses the slides were stained with haematoxylin, rehydrated and coverslipped.

### Whole gene expression analysis

RNA from 9 EW and 11 OS samples was hybridized on Agilent whole human genome microarray (#G4851C, Agilent Technologies), which represents 60k unique human transcripts. Gene expression analysis was conducted on PDXs that were representative of the major clinical variables under study, *i*.*e*. pediatric and adult cases, treated and untreated cases, primary, relapsed and metastatic cases, in all instances selecting a PDX that had yielded an *in vitro* culture (*see* Supplementary Table 1). One-color gene expression was performed according to the manufacturer’s procedure. Briefly, RNA quality was assessed by Agilent Bioanalyzer to have a RIN (RNA integrity number) higher than 7. Labeled cRNA was synthesized from 100 ng of total RNA using the Low Input Quick-Amp Labeling Kit, one color (Agilent Technologies) in the presence of cyanine 3-CTP. Hybridization was performed at 65 °C for 17 hours in a rotating oven. Images at 5 µm (3 µm) resolution were generated by Agilent scanner, Feature Extraction 10.7.3.1 software (Agilent Technologies) was used to obtain the microarray raw-data. Data are deposited in the ArrayExpress database (accession E-MTAB-7568).

### Bioinformatic data analysis

Data were normalized and analyzed using GeneSpring GX v.14.8 software (Agilent Technologies). Data transformation was applied to set all the negative raw values at 1.0, then the quintile normalization was applied. The probes detected in at least one sample were used for statistical analyses. Unsupervised principal component analysis and correlation analysis (Pearson’s correlation) were performed to assess sample similarity and to assess the global gene expression profile of PDX models. Differentially expressed genes were selected to have a ≥2-fold expression difference between matching PDX and primaries and an adjusted p-value ≤ 0.05 at paired t-test, with Benjamini and Hoechberg correction for false positive reduction. Hierarchical clustering was performed for OS samples with GeneSpring clustering tool using the list of differentially expressed genes and the Manhattan correlation as a measure of similarity. Pathway and network analysis of differentially expressed genes was determined using the web-based software MetaCore (GeneGo, Thomson Reuters).

### Sanger analysis of TP53 mutational status and assessment of fusion transcripts

DNA or RNA was extracted from the original tumor or from PDX samples using standard DNAzol or TRIzol procedure (Thermo Fisher Scientific, Foster City, CA, USA). Nucleic acid quality and concentration were evaluated by Nanodrop (Thermo Fisher Scientific). DNA aliquots of 20 µl at the concentration of 12 ng/µl for each sample were used for genotyping analysis of the TP53 mutational status, performed by mean of Sanger Sequencing on an ABI3130xl platform using the BigDye Terminator 3.1 technology (Life Technologies, Carlsbad, CA, USA) amplifying exons together with exon-intron boundaries. The obtained sequence was compared to the NCBI RefSeq (NT_010718) using CodonCode Aligner software (CodonCode Corporation, Centerville, MA, USA) and manual reading. For identification of EWS-ETS fusion transcripts, 500 ng of total RNA was reverse transcribed according the manufacturer’s protocol (High Capacity cDNA Archive Kit, Life Technologies, Carlsbad, CA, USA). cDNA was used as template to amplify EWS-ETS fusion transcripts as previously described^20^. All primer sequences are available upon request.

### *In vivo* therapy of PDX-bearing mice

Freshly obtained fragments of established EW PDX were implanted s.c. in the scapular region of 6–11-week-old immunodeficient male mice. Pharmacological treatments started when tumors reached a volume of 10 mm^3^, *i*.*e*. a mean diameter of 2.7 mm. Animals were randomized to receive two cycles of anti-CD99 diabody C7 (dAbd C7)^21^ peritumorally (Diatheva srl, 1 mg per injection, 5 days/week for 2 consecutive weeks followed by one week of rest) plus irinotecan intraperitoneally (Selleckchem, 0.5 mg/kg, 5 days/week for 1 week, starting after the first week of treatment with dAbd C7); control mice were not treated. Tumor size was measured with calipers; tumor volumes were calculated according to the formula π [√(*a* × *b*)]^3^/6, where *a* = maximal tumor diameter and *b* = tumor diameter perpendicular to *a*. Mouse body weights and tumor volumes were measured at least once a week. Experimental humane endpoint was a tumor maximum volume of 3 cm^3^; as soon as an experimental group (usually untreated controls) overcame this threshold, all other groups were sacrificed to evaluate metastatic spread under comparable conditions. To compare the slopes of tumor growth curves, regression coefficients of linear regressions were calculated and compared pairwise by means of the Prism v 7.03 software (GraphPad Software, San Diego, CA).

### Ethics approval and consent to participate

The collection of human tumor tissue was approved by the ethical committee of the IRCCS Istituto Ortopedico Rizzoli (project #0038254, approval with protocol 0009323) and patient-informed consent forms were obtained for the establishment of PDX models; all methods were performed in accordance with institutional guidelines and Italian law. All animal procedures were done in accordance with European directive 2010/63/UE and Italian Law (DL 26/2014); experimental protocols were reviewed and approved by the institutional animal care and use committee (“Comitato per il Benessere Animale”) of the University of Bologna and by the Italian Ministry of Health with letters 782/2015-PR, 208/2017-PR and 755/2018-PR.

## Results

### Establishment of bone sarcoma PDXs

We implanted in immunodeficient mice 90 primary or metastatic tumor samples, 61 from OS and 29 from EW patients (analytical data are shown in Supplementary Table 1). Successful engraftment was obtained in 36% of OS and 24% of EW samples (Table 1). Of note, extraskeletal OS, which have a poorer prognosis than bone OS^22^, yielded PDX establishment with 100% efficiency, as compared to 30% for bone OS (Table 1).

**Table 1:**
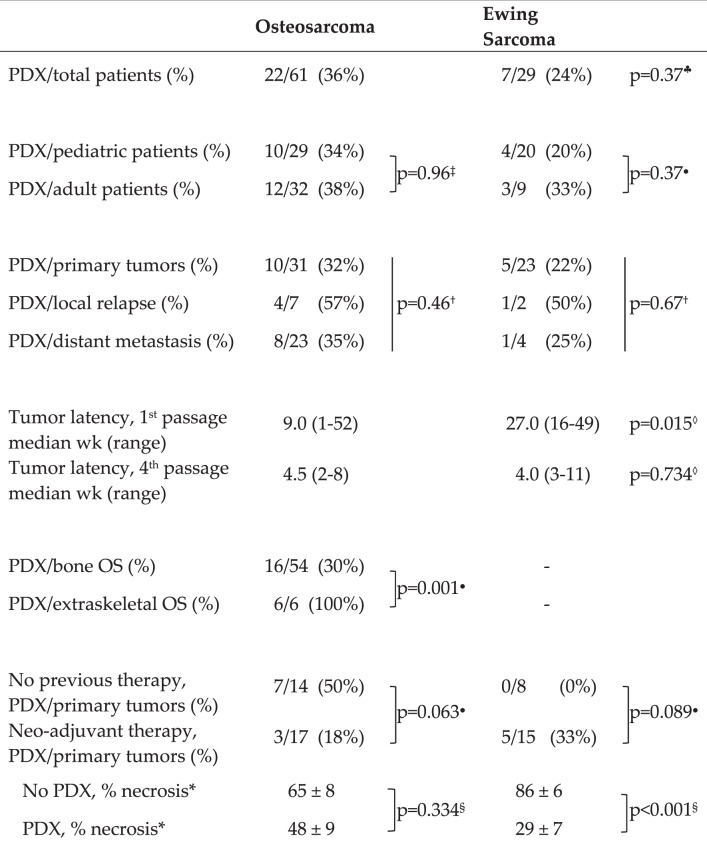
Establishment of bone sarcoma PDX.

^♣^OS *vs* EW, χ^2^ with Yates’s correction.

^‡^χ^2^, Yates’s correction.

^●^Fisher’s exa ct test.

^†^χ^2^, 3 × 2 contingency table.

^◊^OS *vs* EW, Wilcoxon’s non-parametric test.

^§^Student’s *t* test.

*Patient numbers as per “Neo-adjuvant therapy” line above.

No significant difference was observed in engraftment efficiency between OS primary tumors (32%), local relapses (57%) and metastases (35%); analogous conclusions held true for EW, even though limited numbers of relapses and metastases were available (Table 1). In five OS cases we received more than one specimen from the same patient, which included at least one metastatic sample (one primary tumor + metastasis, two cases of local relapse + metastasis, two cases of multiple metastases). Only in one case both specimens yielded a PDX, whereas in four cases one grew as PDX and the other(s) did not (Supplementary Table 1), leading to the conclusion that the ability to engraft was specimen-specific, rather than patient-specific.

Our clinical series contained a mix of pediatric and adult cases, reflecting the cohort of bone sarcoma patients admitted to our Institution, thus we compared their engraftment efficiencies. No significant difference was found between pediatric and adult tumors (Table 1).

After implantation of the surgical sample in mice, the time required for the appearance of a sizeable tumor was highly variable, raging between one week and one year (Table 1). OS patient’s samples generally grew in mice significantly faster than EW (9 *vs*. 27 weeks), however the difference disappeared in subsequent *in vivo* passages, which converged to shorter latency times of the order of 4–5 weeks (Table 1).

We feared that neo-adjuvant cytotoxic therapy, which is frequently administered to OS and EW patients, could jeopardize PDX establishment^23^. We found an opposite trend between the two tumor types. In primary OS the specimens obtained after neoadjuvant therapy gave rise to PDX with a lower efficiency than those obtained in the absence of therapy (Table 1). On the contrary, no EW specimen from untreated patients produced a PDX (Table 1). However, it should be noted that most untreated EW specimens were biopsies, which also failed to yield PDXs (Supplementary Table 1), therefore at least two variables (untreated *vs*. treated and bioptical *vs*. surgical) were at play here, but the numerosity was insufficient for a meaningful stratification of patients.

The level of necrosis induced by therapy was routinely evaluated in all treated patients. In OS patients there was no significant difference in the level of necrosis between cases that gave rise to a PDX and cases which failed to engraft. In contrast, a significant difference was found between those EW patients from which a PDX was obtained, which had a low level of necrosis, and patients which failed to produce a PDX, which had a much higher level of necrosis (Table 1).

A notable difference between our sarcoma series and most carcinoma PDX studies was that we did not observe any human lymphoma development, a frequent event occurring in one-fourth to one-third of mice receiving implants of human carcinomas^23–25^. As human lymphomas of mice implanted with human solid tumors arise from EBV-immortalized human infiltrating lymphocytes growing in the immunodeficient host^25^, the lack of lymphomas in our series could be attributed both to the extreme scarcity of infiltrating lymphocytes in bone sarcomas^26^ and to the young age of patients, which in Western countries have a lower prevalence of EBV positivity than adults^27^.

We took advantage of the availability in our animal facilities of two popular immunodeficient knockout mice, i.e. NSG and RGKO, to directly compare their permissivity to the growth of established human PDX. Overlapping growth rates were observed when equal amounts of the same *in vivo* passage of six OS and three EW PDX were implanted in parallel both in NSG and in RGKO (Supplementary Fig. 1), leading to the conclusion that bone sarcoma PDX established in NSG mice grow equally well in RGKO mice. Other Authors used NSG mice to establish human PDX, then switched to *nude* mice for subsequent passages^28^. The use of a more robust (and in some instances less expensive) mouse host for experiments entailing the use of large numbers of mice could be advantageous.

### Establishment of bone sarcoma cell cultures from clinical samples and from PDXs

We had the opportunity to obtain tumor material also to seed *in vitro* primary cultures from most clinical specimens (73 of 90 cases), thus enabling us to directly compare the two major ways to establish human tumor models^10,17,29^.

We found that the rate of success in establishing primary cell cultures from OS tumor samples was lower than the percentage of successful PDX engraftment in mice, whereas the reverse was observed for EW (Table 2). In both tumor types primary cultures from PDX were established with a significantly higher efficiency than from the original specimen, reaching 100% for EW (Table 2).

**Table 2:**
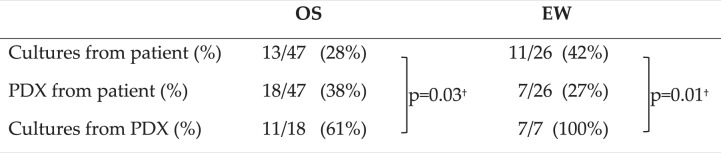
PDX *versus in vitro* cell cultures.

^†^χ^2^, 3 × 2 contingency table.

The overall proportion of clinical specimens yielding primary cultures (24/73, 33%) overlapped that of PDX (25/73, 34%), however we found several discordant cases. On the whole, it can be said that the simultaneous use of both approaches raised the proportion of cases yielding a viable model (either *in vitro* or *in vivo*) from one-third to almost one-half (34/73, 47%). The analysis of concordant and discordant cases showed a strong imbalance between tumor types. All cases which gave rise to a PDX while failing to produce a primary culture were OS (Table 3), thus confirming the propensity of OS to grow *in vivo* rather than *in vitro*.

**Table 3:** Concordance between successful *in vitro* culture from patient specimen and successful PDX, by tumor type*.

Successful culture	Successful PDX	OS + EW cases	OS cases (%)	EW cases (%)
Yes	Yes	15	8 (57%)	7 (43%)
No	No	39	24 (62%)	15 (38%)
Yes	No	9	5 (56%)	4 (44%)
No	Yes	10	10 (100%)	0 (0%)
TOTAL		73	47 (64%)	26 (36%)

*Each line adds to 100%.

### Fidelity and stability of bone sarcoma PDX

To compare PDX models and patient’s tumors we performed histological and molecular profiling at different *in vivo* passages in mice.

A comparative morphological analysis of all xenografts showed that PDX maintained a striking similarity of the histological features with those of the patient’s tumors at least until the third transplant generation (see Fig. 1 for representative pictures). The expression of relevant molecular biomarkers of both tumor types, analyzed in all PDXs, was also faithfully mirrored by PDX, in particular SATB2 and PGP in OS PDX (Fig. 1), CD99 and caveolin 1 in EW PDX (Fig. 1).

Fusion transcripts of EW PDX also mirrored those found in patients (Supplementary Table 2). As TP53 mutations are relatively rare (<10%) in EW^30^, we compared gene sequences of the patient’s sample and of the PDX, because TP53 alterations can arise during the adaptation to *in vitro* culture of human sarcoma cells, resulting in a much higher proportion of cell lines harboring TP53 mutations than actual human tumors^31^. The results showed that *de novo* TP53 mutations did not arise in EW PDX (Supplementary Table 2), further confirming that PDX reliably reproduce the molecular features of the original tumor.

### Gene expression profiling

**Figure 2:**
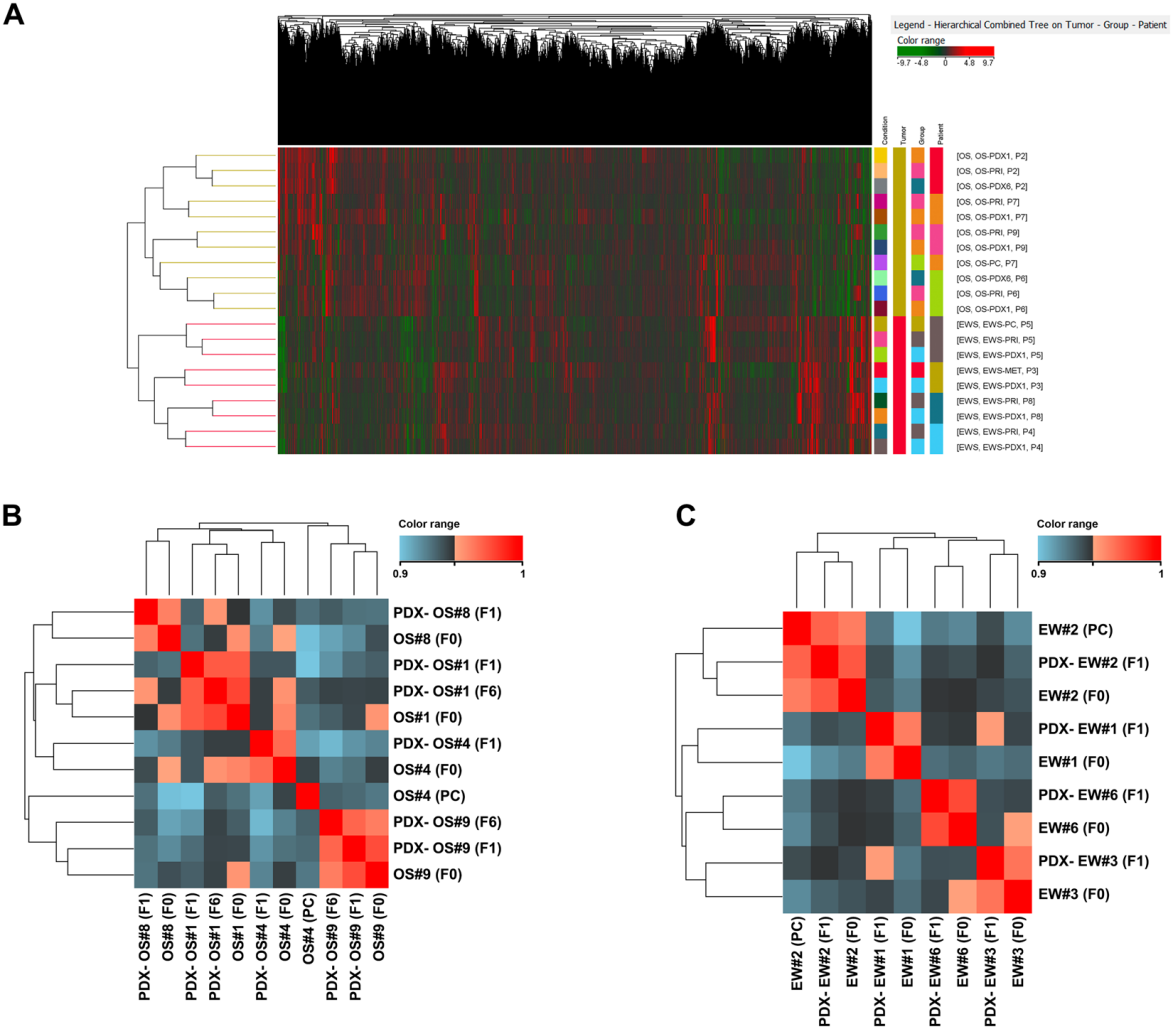
Unsupervised clustering using all (40 K) genes and all specimens (**A**). Correlation between OS (**B**) and EW (**C**) samples calculated using the whole gene expression profile obtained from microarray analysis. Correlation indexes (Pearson’s r) was used to perform the hierarchical clustering of samples (Euclidean distance). Abbreviations: PC, primary *in vitro* culture; F0, patient’s specimen; F1, PDX at first *in vivo* passage; F6, PDX at sixth *in vivo* passage.

To gain a better insight into the similarity of the PDX with the original tumor, we performed a global gene expression correlation analysis between gene expression profiles of primary OS and EW samples and the corresponding PDX. Unsupervised clustering using all (40 k) genes and all samples showed that each tumor histotype formed a separate cluster, and all samples matching tumors and PDXs derived from the same patient clustered together (Fig. 2A). Indeed, the correlation between primary tumors and their PDXs was extremely high for both tumor types (Pearson’s r range r = 0.94–0.96), thus confirming the close resemblance of PDXs to the original human neoplasm. We included in the analysis two OS PDX at the sixth *in vivo* passage, also in these cases we observed a strong correlation with both the original specimen and the first *in vivo* passage (Fig. 2B,C).

**Figure 3:**
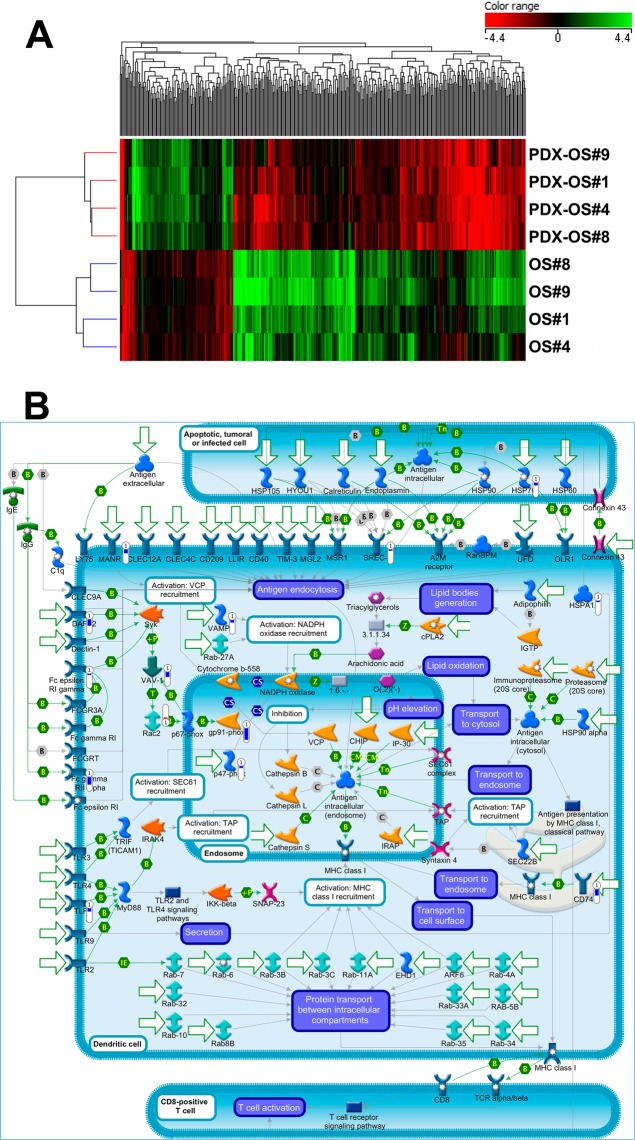
(**A**) Heatmap of OS samples obtained using the list of 397 genes (see Supplementary Table 3) that are differentially expressed (adjusted p < 0.05) between primary tumors and PDX. Genes (columns) and samples (rows) were grouped by hierarchical clustering (Manhattan correlation). High- and low- expression is normalized to the average expression across all samples. (**B**) Map of “Immune response_Antigen presentation by MHC class I” pathway, which is the top scored (lowest *p* value) ma*p* based on Genego pathway enrichment analysis. Experimental data (OS PDX/primary tumor ratio) from microarray experiments are visualized on the map as thermometer-like figures. Significantly upregulated genes show upward, red bars, while down-regulated genes show downward, blue bars.

For EW, the comparison of original patient samples with PDXs did not reveal any significant differentially expressed gene (fold change >2, adjusted p < 0.05 at paired t-test). On the contrary, the comparison between OS primaries and PDX yielded 397 differentially expressed genes (Fig. 3A and Supplementary Table 3).

A Pathway Enrichment Analysis (Metacore software) showed that the genes that differentiate human OS from their PDX belong to immune functional categories (Fig. 3B, Supplementary Tables 4 and 5), in line with the idea that, upon engraftment in the mouse, human leukocytes, which also include lymphocytes, are gradually replaced by leukocytes of the immunodeficient host, lacking T, B and NK cells^32^.

Cell cultures displayed a reduced correlation with the primary tumor if compared to PDX (r = 0.90–0.93, Fig. 2), moreover an unsupervised principal Component Analysis revealed a marked difference between OS- and EW-derived samples, with a major distance within each group for the primary cell line sample (Supplementary Fig. 2), thus indicating that *in vitro* adaptation has a higher impact on the molecular profile than *in vivo* growth^10^. Primary cell cultures appeared less reliable than PDX models, a relevant feature especially in OS, that has a high level of genetic heterogeneity^33,34^.

### Novel chemo-immunotherapeutic combination for the treatment of Ewing sarcoma

**Figure 4:**
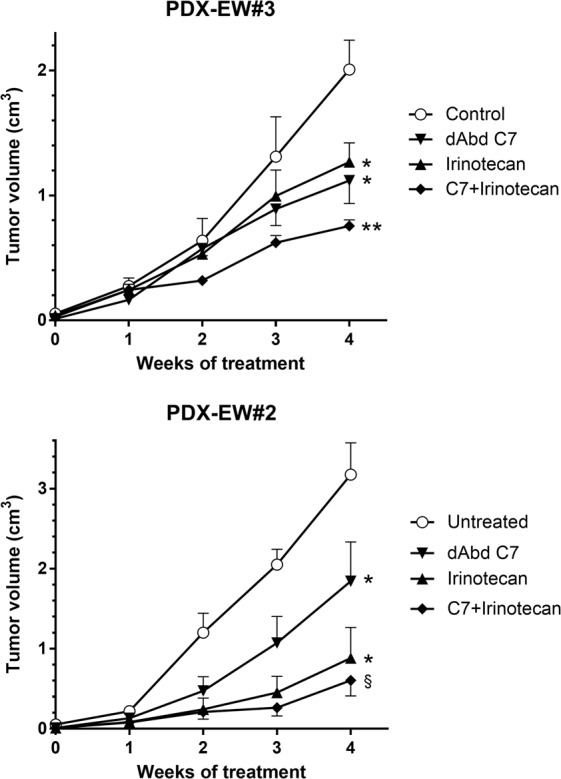
Inhibition of PDX-EW#3 and PDX-EW#2 (as indicated) tumor growth by a combination of anti-CD99 diabody C7 and irinotecan. Groups of 5–6 mice were treated as described under Material and Methods. Statistical comparisons: *slope of linear regression significantly different from untreated, p < 0.05; **slope of linear regression significantly different from all other groups, p < 0.01 at least; ^§^slope of linear regression significantly different from untreated and from C7, p < 0.01 at least, p = 0.17 *versus* irinotecan.

PDX are an ideal model to test therapeutic approaches. We have recently developed dAbd C7, a therapeutic bivalent antibody (diabody) against CD99^21,35,36^, and we were interested in testing combinations with effective drugs in clinical use, such as irinotecan^37^. PDX-EW#3 and PDX-EW#2 were thus treated with dAbd C7, irinotecan or both. While both dAbd C7 and irinotecan effectively hampered tumor growth of both PDXs, the combined treatment provided a definite advantage in comparison to single treatments in PDX-EW#3 (Fig. 4). In PDX-EW#2, which displayed a faster growth rate than PDX-EW#3, the high effectiveness of irinotecan alone was not significantly enhanced by the combined treatment (Fig. 4). Interestingly, we found that 40% (2 of 5) untreated mice bearing PDX-EW#3 tumors had lung metastases, whereas all treated mice were metastasis-free, thus suggesting that this PDX could be further developed to analyze the effect of therapeutic treatments on metastatic spread of EW.

## Discussion

We generated a large panel of bone sarcoma PDX, namely OS and EW, comprising a sizeable number of PDX from pediatric patients, thus creating a powerful tool for future prognostic and sensitivity analyses. All the PDXs described in this work are available for distribution, within the limits of the informed consent of patients, upon the establishment of a standard material transfer agreement.

PDX have been so far successfully established and characterized for many different cancer types, such as colorectal^38,39^, pancreatic^40^, lung^41^, breast^42–44^, ovarian^45,46^ and endometrial cancer^47^. Together with other bone tumor PDX series^17,28,48–53^, the systematic collection of PDX from bone sarcomas, including EW, allows the generation of a wider repository of reliable models for testing drug sensitivity, biomarkers evaluation, or tuning of a personalized therapeutical schedule. A comparison of our series of bone sarcoma PDXs with those recently published by Rainusso *et al*.^53^ and Stewart *et al*.^28^ shows a lower (but not statistically significant) global percentage of engraftment (32% *vs* 54% *vs* 45%, respectively). These differences might result both from differences in the techniques of tumor implantation and from differences in the clinical series. For what concerns the former, Stewart *et al*.^28^ used enzymatic dissociation of clinical samples to obtain a cellular suspension that was then injected orthotopically in mice together with an extracellular matrix preparation, whereas in the paper by Rainusso *et al*.^53^ and in the present work tumor fragments were implanted subcutaneously. Regarding clinical series, both Rainusso *et al*.^53^ and Stewart *et al*.^28^ had a higher percentage of OS than our series, and in all cases OS showed a higher engraftment rate than EW. However, the relative proportion of OS *vs* EW within each clinical series does not seem to explain the higher engraftment rate reported by Rainusso *et al*.^53^ and Stewart *et al*.^28^, because they had higher engraftment rates than ours, in particular within the OS group. A further relevant aspect that might contibute to the observed differences was the different proportion of clinical samples from treated OS patients in each clinical series. As therapeutic treatments and patient’s responses were difficult to compare among the three series, the most straightforward comparison is based on untreated patients. Under this respect, both Rainusso *et al*.^53^ and our series showed comparable engraftment rates (42% *vs* 50%), whereas the series of Stewart *et al*.^28^ was not comparable because it contained only one untreated OS patient. A further element of difference among the three clinical series was the proportion of pediatric *vs* adult cases. Both Rainusso *et al*.^53^ and Stewart *et al*.^28^ only studied pediatric bone sarcomas, whereas our series included a mix of pediatric and adult cases, both among OS and EW. However, this difference should not affect the differences in engraftment, because we found that pediatric and adult bone sarcomas did not significantly differ in the engraftment rates (Table 1).

Two important properties of our PDX panel were revealed by in-depth molecular and morphological studies: a faithful reproduction of the phenotypic features of the human tumor of origin and a considerable stability at least until the 6^th^ *in vivo* generation. A direct comparison of the gene expression profiles of the PDX and of the cell culture obtained from the same patient showed that the PDX better reflected the molecular features of the human tumor than the cell culture. Although the underlying cause of cell lines limited predictive value is not fully understood^10,54,55^, evidence suggests that the process of generating *in vitro* cancer cultures results in major and irreversible alterations of biological properties, including gain and loss of genetic information, alteration in growth and invasion properties, and loss of specific cell populations^10,56,57^.

The faithfulness of preclinical models has been considerably debated in recent times as one of the reasons of the poor translatability of preclinical endeavors into effective therapeutic approaches^14,57,58^. This problem is particularly evident in OS and EW, in which therapeutic options are often inadequate in relapsed disease, but clinical evidence of substantial advancements is lacking^1–3^. The problem is further worsened by the rarity of these two malignancies, which slows down any potential scientific improvement resulting from basic or translational research and results in neglect by big pharma companies. The availability of both pediatric and adult PDX will allow the evaluation of therapeutic regimes tailored to pediatric tumors, which are currently treated with scaled-down protocols originally designed for adult tumors^59,60^. Some recent findings report new drugs sensitivity tested also in EW and OS PDX models^57,61–65^, further sustaining the potential of these models. We investigated here a combination of irinotecan and dAbd C7 treatment in two EW-PDX. Our results suggest that the addition of the anti-CD99 treatment could be beneficial when irinotecan alone is less effective, possibly depending on tumor growth rate. As we tested only two PDX, these conclusions will need to be confirmed in a larger series of preclinical models.

## Conclusion

In conclusion, expandable, shareable and reliable preclinical models are a highly desirable tool for the identification of predictive biomarkers and for the evaluation of effective treatment strategies.

## Supplementary information

Supplementary files

## Data Availability

The datasets generated during and/or analysed during the current study are available in the ArrayExpress repository (accession E-MTAB-7568).
