# Supplementary material for: Bone sarcoma patient-derived xenografts are faithful and stable preclinical models for molecular and therapeutic investigations
Source: Sci Rep. 2019 Aug 21;9:12174. doi: 10.1038/s41598-019-48634-y (PMC6704066; doi:10.1038/s41598-019-48634-y)

Bone sarcoma patient-derived xenografts are faithful and stable preclinical models for molecular and therapeutic investigations

Patrizia Nanni, Lorena Landuzzi, Maria Cristina Manara, Alberto Righi, Giordano Nicoletti, Camilla Cristalli, Michela Pasello, Alessandro Parra, Marianna Carrabotta, Manuela Ferracin, Arianna Palladini, Marianna L. Ianzano, Veronica Giusti, Francesca Ruzzi, Mauro Magnani, Davide Maria Donati, Piero Picci, Pier-Luigi Lollini and Katia Scotlandi

Description of Supplementary files

Supplementary Table 1. Patients characteristics, PDX and cell cultures

Supplementary Table 2. Fusion transcripts and p53 status of EW PDX

Supplementary Table 3. List of genes differentially expressed in OS PDX *vs.* clinical sample

Supplementary Table 4. Most relevant networks involving the genes that are deregulated in PDX *vs.* primary OS tumors

Supplementary Table 5. Enrichment by Pathway Maps.

Supplementary Figure 1. Comparison of PDX growth in two immunodeficient mouse strains.

Supplementary Figure 2. Unsupervised Principal Component Analysis of OS and EW including clinical samples, PDX and *in vitro* cultures.

Supplementary Table 1. Patients characteristics, PDX and cell cultures

Specimen code	Patient age (years)	Patient sex	Tumor histotype	Specimen origin (primary tumor / local relapse / distant metastasis*)	Notes	Multiple specimens from same patient**	Specimen type	Post-chemo therapy	PDX growth	PDX name	*In vitro* growth from specimen	*In vitro* growth from PDX
R001	18	M	OS	Primary		1	Biopsy	NO	YES	PDX-OS#1	nd	YES
R002	49	F	EW	Metastasis			Surgery	YES	YES	PDX-EW#1	YES	YES
R003	37	M	OS	Relapse	Extraskeletal		Surgery	NO	YES	PDX-OS#3	YES	NO
R004	16	M	OS	Metastasis		2	Surgery	YES	NO		YES	nd
R005	19	M	OS	Primary			Surgery	YES	NO		NO	nd
R006	11	F	OS	Primary			Biopsy	NO	YES	PDX-OS#4	YES	YES
R007	20	F	OS	Metastasis			Surgery	YES	NO		YES	nd
R008	49	F	OS	Metastasis			Surgery	YES	NO		YES	nd
R009	27	M	OS	Relapse			Surgery	NO	NO		YES	nd
R010	8	M	EW	Primary			Surgery	YES	NO		NO	nd
R011	7	F	EW	Primary			Surgery	YES	YES	PDX-EW#6	YES	YES
R012	43	M	OS	Primary			Surgery	YES	NO		NO	nd
R013	35	M	OS	Metastasis			Surgery	YES	NO		NO	nd
R014	38	M	EW	Relapse			Surgery	NO	YES	PDX-EW#2	YES	YES
R015	68	M	OS	Relapse	Extraskeletal		Surgery	NO	YES	PDX-OS#5	NO	NO
R016	52	M	OS	Metastasis			Surgery	YES	NO		YES	nd
R017	12	F	OS	Primary			Surgery	YES	YES	PDX-OS#2	NO	YES
R018	49	F	OS	Primary	Extraskeletal		Surgery	YES	YES	PDX-OS#6	YES	YES
R019	38	M	OS	Relapse	Extraskeletal		Surgery	YES	YES	PDX-OS#7	YES	YES
R020	17	M	OS	Primary		3	Biopsy	NO	YES	PDX-OS#8	NO	YES
R021	71	F	OS	Primary	Extraskeletal		Surgery	NO	YES	PDX-OS#9	NO	YES
R022	49	F	OS	Primary			Surgery	YES	NO		NO	nd
R023	36	F	OS	Relapse	Extraskeletal	4	Surgery	NO	YES	PDX-OS#10	NO	NO
R024	36	F	OS	Metastasis		4	Surgery	YES	NO		NO	nd
R025	16	F	EW	Relapse			Surgery	YES	NO		NO	nd
R026	28	M	OS	Relapse		7	Surgery	YES	NO		NO	nd

R027	7	M	OS	Primary			Biopsy	NO	NO		NO	nd
R028	14	M	EW	Primary			Surgery	YES	YES	PDX-EW#3	YES	YES
R029	45	M	EW	Metastasis			Surgery	NO	NO		YES	nd
R030	24	M	OS	Metastasis			Surgery	YES	NO		NO	nd
R031	17	M	OS	Primary		3	Surgery	YES	NO		NO	nd
R032	14	F	EW	Primary			Surgery	YES	NO		NO	nd
R033	14	M	EW	Primary			Surgery	YES	NO		NO	nd
R034	15	M	EW	Primary		5	Biopsy	YES	NO		NO	nd
R035	14	F	OS	Primary			Surgery	YES	NO		NO	nd
R036	22	M	OS	Primary			Biopsy	NO	YES	PDX-OS#11	NO	YES
R037	13	M	EW	Primary			Surgery	NO	NO		YES	nd
R038	43	F	OS	Metastasis			Surgery	YES	YES	PDX-OS#14	NO	YES
R039	26	M	EW	Metastasis			Surgery	YES	NO		NO	nd
R040	17	M	OS	Metastasis		2	Surgery	YES	NO		nd	nd
R041	12	M	EW	Primary		6	Biopsy	NO	NO		NO	nd
R042	13	M	EW	Primary			Biopsy	NO	NO		NO	nd
R043	11	M	EW	Primary			Biopsy	NO	NO		NO	nd
R044	17	M	OS	Metastasis		2	Surgery	YES	NO		NO	nd
R045	27	F	OS	Primary			Surgery	NO	NO		NO	nd
R046	28	M	OS	Metastasis		7	Surgery	YES	YES	PDX-OS#15	nd	YES
R047	10	F	OS	Primary	Periosteal		Biopsy	NO	NO		NO	nd
R048	26	M	OS	Metastasis			Surgery	YES	NO		NO	nd
R049	51	F	OS	Metastasis			Surgery	YES	NO		NO	nd
R050	18	F	OS	Metastasis			Surgery	YES	NO		nd	nd
R051	15	M	EW	Primary		5	Surgery	YES	NO		NO	nd
R052	19	M	OS	Metastasis		1	Surgery	YES	NO		NO	nd
R053	11	M	EW	Primary		6	Surgery	YES	NO		nd	nd
R054	10	F	OS	Metastasis		8	Surgery	YES	YES	PDX-OS#16	nd	YES
R055	36	F	OS	Metastasis			Surgery	YES	YES	PDX-OS#17	NO	NO
R056	9	M	EW	Primary	Askin tumor		Surgery	YES	NO		NO	nd
R057	21	M	EW	Primary	Askin tumor		Surgery	YES	NO		YES	nd
R058	44	M	EW	Metastasis			Surgery	YES	NO		NO	nd
R059	17	M	OS	Primary			Surgery	NO	NO		NO	nd
R060	50	F	OS	Primary			Surgery	YES	NO		NO	nd

R061	5	F	OS	Primary			Biopsy	NO	YES	PDX-OS#21	YES	YES
R062	10	F	OS	Metastasis		8	Surgery	YES	YES	PDX-OS#18	NO	NO
R063	17	F	EW	Primary			Surgery	YES	YES	PDX-EW#4	YES	YES
R064	21	M	OS	Primary			Surgery	YES	NO		nd	nd
R065	55	M	OS	Metastasis			Surgery	YES	NO		NO	nd
R066	30	M	OS	Metastasis			Surgery	NO	YES	PDX-OS#19	NO	YES
R067	20	M	OS	Primary			Biopsy	NO	NO		NO	nd
R068	11	F	OS	Primary			Surgery	YES	NO		NO	nd
R069	8	M	OS	Primary			Surgery	YES	NO		NO	nd
R070	21	M	EW	Primary			Biopsy	NO	NO		YES	nd
R071	15	M	EW	Primary			Surgery	YES	NO		NO	nd
R072	11	F	OS	Primary		9	Biopsy	NO	NO		nd	nd
R073	25	M	EW	Primary			Surgery	YES	YES	PDX-EW#5	YES	YES
R074	5	F	OS	Primary			Surgery	YES	NO		NO	nd
R075	18	M	OS	Primary			Surgery	YES	NO		nd	nd
R076	13	F	EW	Primary			Surgery	NO	NO		NO	nd
R077	11	F	OS	Primary			Surgery	YES	NO		nd	nd
R078	20	M	OS	Metastasis			Surgery	YES	YES	PDX-OS#20	YES	NO
R079	12	F	OS	Primary			Biopsy	NO	NO		nd	nd
R080	15	M	OS	Primary			Surgery	YES	YES	PDX-OS#12	YES	NO
R081	13	M	OS	Primary			Biopsy	NO	YES	PDX-OS#13	YES	YES
R082	15	M	OS	Metastasis			Surgery	YES	NO		nd	nd
R083	11	F	OS	Primary		9	Surgery	YES	NO		NO	nd
R084	16	M	EW	Primary			Biopsy	NO	NO		nd	nd
R085	18	M	OS	Metastasis		2	Surgery	YES	YES	PDX-OS#23	nd	YES
R086	10	F	OS	Relapse			Biopsy	YES	NO		nd	nd
R087	39	M	EW	Primary			Surgery	YES	NO		nd	nd
R088	20	M	OS	Primary			Surgery	YES	NO		nd	nd
R089	14	F	EW	Primary	Askin tumor		Surgery	NO	NO		NO	nd
R090	2	M	EW	Primary	Askin tumor		Surgery	YES	YES	PDX-EW#7	YES	YES

*All metastatic specimens were from lung metastases, except R029, from a bone metastasis
**Identical numbers denote multiple specimens from the same patient
nd, not done

Supplementary Table 2. Fusion transcripts and p53 status of EW PDX

PDX name	Fusion transcript		p53 status	
	**Patient**	**PDX***	**Patient**	**PDX***
EW#1	EWS-FLI1 Type 1	EWS-FLI1 Type 1	Wild-type	Wild-type
EW#2	EWS-FLI1 complex type	EWS-FLI1 complex type	Wild-type	Wild-type
EW#3	EWS-ERG	EWS- ERG	Wild-type	Wild-type
EW#4	EWS-FLI1 Type 1	EWS-FLI1 Type 1	Wild-type	Wild-type
EW#5	EWS-FLI1 Type 2	EWS-FLI1 Type 2	Wild-type	Wild-type
EW#6	EWS-FLI1 Type 1	EWS-FLI1 Type 1	nd†	nd†

*First *in vivo* passage

†nd, not done

Supplementary Table 3. List of genes differentially expressed in OS PDX *vs.* clinical sample

Notes : Created from Advanced Analysis operation: significance Analysis.
#Entitylist : Fold change >= 2.0 OS
#Interpretation : Tumor - Group OS
#Experiment: Sarcomas and PDX - quantile
#corrected p-value cut-off:0.05
#Fold change cut-off:2.0
#Selected Test : Moderated T-Test
#p-value computation: Asymptotic
#Multiple Testing Correction: Benjamini-Hochberg
Technology : Agilent.SingleColor.72363
Owner : gxuser
Created On : Fri Oct 21 17:05:18 CEST 2016

ProbeName	Fold change	Regulation	p (Corr)	p	[OS, OS-PRI] (normalized)	[OS, OS-PDX1] (normalized)	[OS, OS-PDX6] (normalized)	GeneSymbol	Description	GenbankAccession
A_23_P204847	-32.827225	down	3.41E-02	2.655E-03	9.442151	4.40533	2.1022692	LCP1	Homo sapiens lymphocyte cytosolic protein 1 (L-plastin) (LCP1), mRNA [NM_002298]	NM_002298
A_33_P3364811	-17.480547	down	1.17E-02	3.494E-04	5.9815836	1.8539052	1.8470192	PTPRC	Homo sapiens protein tyrosine phosphatase, receptor type, C (PTPRC), transcript variant 1, mRNA [NM_002838]	NM_002838
A_24_P32935	-3.6771917	down	4.70E-02	4.444E-03	6.6413274	4.762723	3.5856256	FOLR2	Homo sapiens folate receptor 2 (fetal) (FOLR2), transcript variant 1, mRNA [NM_000803]	NM_000803
A_24_P50245	-6.324459	down	3.41E-02	2.642E-03	8.16873	5.5077877	4.8993483	HLA-DMA	Homo sapiens major histocompatibility complex, class II, DM alpha (HLA-DMA), mRNA [NM_006120]	NM_006120
A_33_P3286157	-4.418821	down	3.09E-02	2.127E-03	9.246864	7.103203	7.12125	TNFRSF4	Homo sapiens tumor necrosis factor receptor superfamily, member 4 (TNFRSF4), mRNA [NM_003327]	NM_003327
A_23_P161076	-38.950096	down	8.77E-03	2.280E-04	7.199453	1.9158978	1.7511885	CD2	Homo sapiens CD2 molecule (CD2), mRNA [NM_001767]	NM_001767
A_33_P3215768	-24.098427	down	7.79E-03	1.867E-04	6.9264827	2.3356159	2.8915195	GALNT6	Homo sapiens polypeptide N-acetylgalactosaminyltransferase 6 (GALNT6), mRNA [NM_007210]	NM_007210
A_24_P937325	-3.6299024	down	4.39E-02	3.836E-03	3.6232808	1.76335	1.782822	FGD4	FYVE, RhoGEF and PH domain containing 4 [Source:HGNC Symbol;Acc:HGNC:19125] [ENST00000472289]	AL713762
A_23_P324813	-7.8121295	down	2.55E-02	1.569E-03	7.460657	4.494941	4.8674817	BCL6B	Homo sapiens B-cell CLL/lymphoma 6, member B (BCL6B), mRNA [NM_181844]	NM_181844
A_19_P00317360	-5.8997593	down	1.61E-03	1.194E-05	4.367965	1.807309	1.8436997	ATP2C1	Homo sapiens ATPase, Ca++ transporting, type 2C, member 1 (ATP2C1), transcript variant 8, mRNA [NM_001199182]	NM_001199182
A_23_P111132	-5.0537076	down	1.61E-02	6.463E-04	12.6707945	10.333452	10.73757	HSPA1A	Homo sapiens heat shock 70kDa protein 1A (HSPA1A), mRNA [NM_005345]	NM_005345
A_23_P102000	-18.995161	down	1.93E-02	8.982E-04	10.495874	6.2483144	2.584436	CXCR4	Homo sapiens chemokine (C-X-C motif) receptor 4 (CXCR4), transcript variant 1, mRNA [NM_001008540]	NM_001008540
A_23_P346093	-7.2225404	down	3.20E-02	2.364E-03	6.2370424	3.384536	4.8317556	TMC8	Homo sapiens transmembrane channel-like 8 (TMC8), mRNA [NM_152468]	NM_152468
A_24_P153568	-22.611359	down	4.00E-02	3.355E-03	7.9167542	3.4177783	1.8121264	MPEG1	Homo sapiens macrophage expressed 1 (MPEG1), mRNA [NM_001039396]	NM_001039396
A_24_P237443	-14.832554	down	2.31E-03	2.707E-05	5.6262846	1.7355895	1.7281557	SASH3	Homo sapiens SAM and SH3 domain containing 3 (SASH3), mRNA [NM_018990]	NM_018990
A_33_P3296181	-3.749955	down	2.32E-02	1.256E-03	5.513463	3.6065898	3.6167006	CCL3L3	Homo sapiens chemokine (C-C motif) ligand 3-like 3 (CCL3L3), mRNA [NM_001001437]	NM_001001437
A_32_P356316	-40.149208	down	2.25E-02	1.160E-03	8.290367	2.9630675	1.7372084	HLA-DOA	Homo sapiens major histocompatibility complex, class II, DO alpha (HLA-DOA), mRNA [NM_002119]	NM_002119
A_33_P3369393	-7.871324	down	2.32E-02	1.272E-03	7.5205135	4.543907	4.50961	NCF1	Homo sapiens neutrophil cytosolic factor 1 (NCF1), mRNA [NM_000265]	NM_000265
A_23_P3014	-27.93256	down	1.81E-03	1.893E-05	6.8613033	2.0574276	1.9349339	RNASE6	Homo sapiens ribonuclease, RNase A family, k6 (RNASE6), mRNA [NM_005615]	NM_005615
A_24_P40551	-9.3017435	down	3.99E-03	6.810E-05	5.21972	2.0022187	1.788909	BEX4	Homo sapiens brain expressed, X-linked 4 (BEX4), transcript variant 2, mRNA [NM_001080425]	NM_001080425
A_24_P319364	-52.32558	down	3.75E-03	6.123E-05	7.938652	2.2292075	1.7852266	F11R	Homo sapiens F11 receptor (F11R), mRNA [NM_016946]	NM_016946
A_23_P7313	-45.404552	down	7.85E-03	1.933E-04	12.508218	7.003453	2.767918	SPP1	Homo sapiens secreted phosphoprotein 1 (SPP1), transcript variant 1, mRNA [NM_001040058]	NM_001040058
A_33_P3365432	-11.647932	down	1.92E-02	8.899E-04	6.2003665	2.6583645	2.4588814	NCF4	Homo sapiens neutrophil cytosolic factor 4, 40kDa (NCF4), transcript variant 1, mRNA [NM_000631]	NM_000631
A_33_P3229402	-24.10832	down	1.91E-02	8.769E-04	6.8765974	2.285138	2.3170085	PECAM1	Homo sapiens platelet/endothelial cell adhesion molecule 1 (PECAM1), mRNA [NM_000442]	NM_000442
A_23_P85240	-12.127025	down	1.97E-02	9.289E-04	5.912547	2.3123934	2.0178282	TLR7	Homo sapiens toll-like receptor 7 (TLR7), mRNA [NM_016562]	NM_016562
A_23_P91414	-13.814562	down	6.52E-03	1.446E-04	5.592788	1.8046703	2.1999748	CCM2L	Homo sapiens cerebral cavernous malformation 2-like (CCM2L), mRNA [NM_080625]	NM_080625
A_23_P10506	-21.835701	down	2.17E-02	1.089E-03	7.068879	2.6202621	1.8327141	HPGDS	Homo sapiens hematopoietic prostaglandin D synthase (HPGDS), mRNA [NM_014485]	NM_014485
A_23_P166297	-8.040049	down	3.49E-02	2.752E-03	6.352495	3.345291	3.0164967	ABCG1	Homo sapiens ATP-binding cassette, sub-family G (WHITE), member 1 (ABCG1), transcript variant 5, mRNA [NM_207627]	NM_207627
A_23_P121480	-12.75118	down	5.69E-03	1.176E-04	6.256212	2.5836535	2.0513625	CD200	Homo sapiens CD200 molecule (CD200), transcript variant 2, mRNA [NM_001004196]	NM_001004196
A_32_P181077	-17.802723	down	3.59E-03	5.496E-05	6.2487783	2.0947526	1.9579582	DOCK8	Homo sapiens dedicator of cytokinesis 8 (DOCK8), transcript variant 1, mRNA [NM_203447]	NM_203447
A_23_P21495	-5.4419465	down	1.93E-02	9.049E-04	9.956417	7.5122943	5.0542536	FCGBP	Homo sapiens Fc fragment of IgG binding protein (FCGBP), mRNA [NM_003890]	NM_003890
A_33_P3424217	-29.82521	down	2.47E-03	3.259E-05	7.602979	2.704519	1.8369366	HLA-DQB1	Homo sapiens major histocompatibility complex, class II, DQ beta 1 (HLA-DQB1), transcript variant 3, mRNA [NM_001243962]	NM_001243962
A_33_P3218975	-11.902355	down	7.84E-03	1.899E-04	5.5534506	1.9802754	1.741519	ENTPD1	Homo sapiens ectonucleoside triphosphate diphosphohydrolase 1 (ENTPD1), transcript variant 1, mRNA [NM_001776]	NM_001776
A_23_P110791	-22.033161	down	3.33E-02	2.501E-03	11.546739	7.085134	6.916711	CSF1R	Homo sapiens colony stimulating factor 1 receptor (CSF1R), transcript variant 1, mRNA [NM_005211]	NM_005211
A_23_P203376	-6.4948006	down	4.71E-02	4.506E-03	4.5579133	1.858628	1.8354985	MS4A6A	Homo sapiens membrane-spanning 4-domains, subfamily A, member 6A (MS4A6A), transcript variant 1, mRNA [NM_152852]	NM_152852
A_33_P3215948	-34.317272	down	1.21E-02	3.849E-04	7.9412894	2.8404264	2.5889754	MPZL2	Homo sapiens myelin protein zero-like 2 (MPZL2), transcript variant 1, mRNA [NM_005797]	NM_005797

A_33_P3412438	-3.5580637	down	3.99E-02	3.306E-03	6.25051	4.419418	5.4471207			
A_22_P00007787	-2.1363099	down	4.47E-02	4.084E-03	3.3242722	2.2291512	3.2977633	LOC101928445	Homo sapiens uncharacterized LOC101928445 (LOC101928445), long non-coding RNA [NR_110560]	NR_110560
A_33_P3215883	-21.79865	down	2.33E-02	1.329E-03	6.541357	2.0951903	1.7966506	FLT1	Homo sapiens fms-related tyrosine kinase 1 (FLT1), transcript variant 1, mRNA [NM_002019]	NM_002019
A_33_P3316273	-39.538372	down	3.53E-02	2.788E-03	10.692498	5.3873167	1.9101012	CCL3	Homo sapiens chemokine (C-C motif) ligand 3 (CCL3), mRNA [NM_002983]	NM_002983
A_22_P00001150	-4.9014506	down	4.89E-02	4.823E-03	4.603488	2.3102791	2.3225336	PCED1B-AS1	Homo sapiens PCED1B antisense RNA 1 (PCED1B-AS1), long non-coding RNA [NR_026544]	NR_026544
A_24_P339944	-10.955846	down	4.04E-02	3.397E-03	6.828881	3.3752518	4.0652614	PDGFB	Homo sapiens platelet-derived growth factor beta polypeptide (PDGFB), transcript variant 1, mRNA [NM_002608]	NM_002608
A_23_P145631	-26.285011	down	1.61E-03	1.159E-05	6.9543705	2.2382019	1.774046	GIMAP6	Homo sapiens GTPase, IMAP family member 6 (GIMAP6), transcript variant 1, mRNA [NM_024711]	NM_024711
A_24_P224727	-9.088336	down	4.57E-02	4.276E-03	7.388281	4.2042646	5.330721	CEBPA	Homo sapiens CCAAT/enhancer binding protein (C/EBP), alpha (CEBPA), transcript variant 1, mRNA [NM_004364]	NM_004364
A_23_P203173	-65.764755	down	7.85E-03	1.943E-04	9.004591	2.9653485	1.7895797	IL10RA	Homo sapiens interleukin 10 receptor, alpha (IL10RA), transcript variant 1, mRNA [NM_001558]	NM_001558
A_33_P3312466	-10.745551	down	4.52E-02	4.162E-03	5.5664673	2.1407998	1.9421599	BTNL9	Homo sapiens butyrophilin-like 9 (BTNL9), mRNA [NM_152547]	NM_152547
A_33_P3285540	-111.41531	down	1.22E-02	3.966E-04	9.50794	2.7081363	4.126291	CLDN5	Homo sapiens claudin 5 (CLDN5), transcript variant 1, mRNA [NM_001130861]	NM_001130861
A_23_P252471	-33.280064	down	4.99E-02	5.055E-03	9.95705	4.900464	4.586234	PECAM1	Homo sapiens platelet/endothelial cell adhesion molecule 1 (PECAM1), mRNA [NM_000442]	NM_000442
A_23_P118025	-9.382408	down	1.57E-03	1.007E-05	5.080383	1.8504245	1.8267515	DPEP2	Homo sapiens dipeptidase 2 (DPEP2), mRNA [NM_022355]	NM_022355
A_23_P135990	-15.135195	down	1.46E-02	5.669E-04	5.800604	1.8807685	2.2454143	SLCO2A1	Homo sapiens solute carrier organic anion transporter family, member 2A1 (SLCO2A1), mRNA [NM_005630]	NM_005630
A_24_P406132	-5.1868215	down	4.73E-02	4.531E-03	5.5795565	3.2047057	3.9753354	MAPK13	Homo sapiens mitogen-activated protein kinase 13 (MAPK13), transcript variant 1, mRNA [NM_002754]	NM_002754
A_23_P73429	-20.232304	down	2.36E-02	1.378E-03	10.076151	5.737562	8.502817	HCLS1	Homo sapiens hematopoietic cell-specific Lyn substrate 1 (HCLS1), transcript variant 1, mRNA [NM_005335]	NM_005335
A_23_P216340	-5.8616786	down	3.98E-02	3.288E-03	8.536206	5.9848924	5.526022	SLA	Homo sapiens Src-like-adaptor (SLA), transcript variant 1, mRNA [NM_001045556]	NM_001045556
A_23_P427023	-27.40517	down	1.23E-02	4.050E-04	7.2497663	2.47339	2.6647005	GIMAP1	Homo sapiens GTPase, IMAP family member 1 (GIMAP1), mRNA [NM_130759]	NM_130759
A_33_P3352098	-10.211405	down	2.51E-02	1.538E-03	8.9233465	5.571237	4.872101	MS4A7	Homo sapiens membrane-spanning 4-domains, subfamily A, member 7 (MS4A7), transcript variant 1, mRNA [NM_021201]	NM_021201
A_23_P340019	-10.67047	down	6.15E-03	1.333E-04	5.3805275	1.9649756	1.7867563	NLRC3	Homo sapiens NLR family, CARD domain containing 3 (NLRC3), transcript variant 1, mRNA [NM_178844]	NM_178844
A_22_P00005437	-5.7419763	down	4.71E-02	4.487E-03	4.4812818	1.9597344	2.834795	lnc-DUOX2-2	LNCipedia lincRNA (lnc-DUOX2-2), lincRNA [lnc-DUOX2-2:1]	
A_23_P90626	-19.090994	down	1.25E-02	4.166E-04	6.081032	1.8262115	1.8083856	CYTIP	Homo sapiens cytohesin 1 interacting protein (CYTIP), mRNA [NM_004288]	NM_004288
A_23_P33723	-35.007713	down	3.15E-02	2.242E-03	9.475334	4.345733	3.4004138	CD163	Homo sapiens CD163 molecule (CD163), transcript variant 1, mRNA [NM_004244]	NM_004244
A_23_P368711	-4.390435	down	3.71E-03	5.867E-05	5.1489315	3.0145676	3.2850416	LILRA6	Homo sapiens leukocyte immunoglobulin-like receptor, subfamily A (with TM domain), member 6 (LILRA6), transcript variant 1, mRNA [NM_024318]	NM_024318
A_33_P3212232	-9.21279	down	5.29E-03	1.061E-04	5.500349	2.296711	2.4431298	MPEG1	Homo sapiens macrophage expressed 1 (MPEG1), mRNA [NM_001039396]	NM_001039396
A_23_P86283	-18.427406	down	1.17E-02	3.455E-04	8.366631	4.1628494	3.8952055	LAPTM5	Homo sapiens lysosomal protein transmembrane 5 (LAPTM5), mRNA [NM_006762]	NM_006762
A_23_P11201	-11.857812	down	3.56E-02	2.819E-03	5.755698	2.1879323	2.6044235	GPR34	Homo sapiens G protein-coupled receptor 34 (GPR34), transcript variant 4, mRNA [NM_001097579]	NM_001097579
A_23_P85800	-60.256935	down	1.28E-02	4.444E-04	9.51067	3.5976145	1.9960341	CD52	Homo sapiens CD52 molecule (CD52), mRNA [NM_001803]	NM_001803
A_33_P3309556	-5.0198975	down	4.74E-02	4.554E-03	5.0274205	2.6997626	2.8177948	PTPRE	Homo sapiens protein tyrosine phosphatase, receptor type, E (PTPRE), transcript variant 1, mRNA [NM_006504]	NM_006504
A_23_P47034	-6.230628	down	1.46E-02	5.683E-04	5.1008277	2.46145	4.9880705	HHEX	Homo sapiens hematopoietically expressed homeobox (HHEX), mRNA [NM_002729]	NM_002729
A_23_P82775	-35.308437	down	1.62E-02	6.675E-04	7.070899	1.9289582	1.8604704	SOX17	Homo sapiens SRY (sex determining region Y)-box 17 (SOX17), mRNA [NM_022454]	NM_022454
A_33_P3273884	-16.116735	down	3.33E-02	2.515E-03	6.284051	2.2735631	1.7338192	HLA-DQA1	Homo sapiens major histocompatibility complex, class II, DQ alpha 1 (HLA-DQA1), mRNA [NM_002122]	NM_002122
A_24_P120251	-15.607418	down	1.26E-02	4.246E-04	5.9952555	2.0310955	1.8941857	TM4SF18	Homo sapiens transmembrane 4 L six family member 18 (TM4SF18), transcript variant 1, mRNA [NM_138786]	NM_138786
A_33_P3284508	-14.992103	down	1.15E-02	3.383E-04	12.427656	8.521525	8.733506	CD14	Homo sapiens CD14 molecule (CD14), transcript variant 3, mRNA [NM_001174104]	NM_001174104
A_24_P215653	-15.963263	down	1.20E-02	3.748E-04	6.5014515	2.504768	2.5678906	CLEC14A	Homo sapiens C-type lectin domain family 14, member A (CLEC14A), mRNA [NM_175060]	NM_175060
A_23_P63371	-3.126287	down	4.53E-02	4.202E-03	3.780581	2.1361308	2.5088134	TAL1	Homo sapiens T-cell acute lymphocytic leukemia 1 (TAL1), transcript variant 1, mRNA [NM_003189]	NM_003189
A_24_P340128	-30.798923	down	1.08E-03	3.292E-06	6.6979113	1.7531031	2.1640775	P2RY8	Homo sapiens purinergic receptor P2Y, G-protein coupled, 8 (P2RY8), mRNA [NM_178129]	NM_178129
A_33_P3409062	-26.833282	down	1.79E-02	8.031E-04	12.342063	7.5961113	5.576063	TYROBP	Homo sapiens TYRO protein tyrosine kinase binding protein (TYROBP), transcript variant 1, mRNA [NM_003332]	NM_003332
A_33_P3218980	-7.995942	down	3.09E-02	2.155E-03	5.120029	2.120761	1.7354077	ENTPD1	Homo sapiens ectonucleoside triphosphate diphosphohydrolase 1 (ENTPD1), transcript variant 1, mRNA [NM_001776]	NM_001776
A_23_P38959	-21.37979	down	1.74E-03	1.479E-05	7.0462885	2.628113	1.7360837	VAV1	Homo sapiens vav 1 guanine nucleotide exchange factor (VAV1), transcript variant 1, mRNA [NM_005428]	NM_005428
A_24_P365767	-48.22627	down	2.32E-02	1.279E-03	8.587856	2.9961088	2.293069	CYBB	Homo sapiens cytochrome b-245, beta polypeptide (CYBB), mRNA [NM_000397]	NM_000397
A_33_P3248265	-13.368791	down	2.91E-02	1.982E-03	5.6318483	1.8910512	3.0493646	LTB	Homo sapiens lymphotoxin beta (TNF superfamily, member 3) (LTB), transcript variant 1, mRNA [NM_002341]	NM_002341
A_24_P944253	-7.781011	down	1.28E-03	6.149E-06	4.822876	1.8629185	1.7340091	KLHL6	Homo sapiens kelch-like family member 6 (KLHL6), mRNA [NM_130446]	NM_130446
A_24_P12397	-13.526044	down	3.38E-02	2.571E-03	5.7231894	1.9655215	1.7461956	TREM2	Homo sapiens triggering receptor expressed on myeloid cells 2 (TREM2), transcript variant 1, mRNA [NM_018965]	NM_018965
A_23_P36120	-54.779446	down	2.79E-03	3.980E-05	7.5967197	1.8211569	2.2601695	MS4A6A	Homo sapiens membrane-spanning 4-domains, subfamily A, member 6A (MS4A6A), transcript variant 2, mRNA [NM_022349]	NM_022349
A_24_P362193	-7.40628	down	4.47E-02	3.993E-03	4.959797	2.0710478	1.9313121	CD84	Homo sapiens CD84 molecule (CD84), transcript variant 2, mRNA [NM_003874]	NM_003874
A_22_P00002164	-4.391826	down	1.18E-02	3.599E-04	3.8775008	1.7426797	1.7382627	RPS6KA2-IT1	Homo sapiens RPS6KA2 intronic transcript 1 (non-protein coding) (RPS6KA2-IT1), long non-coding RNA [NR_046793]	NR_046793
A_23_P47709	-21.116518	down	3.18E-02	2.326E-03	9.583854	5.1835537	4.561588	FOLR2	Homo sapiens folate receptor 2 (fetal) (FOLR2), transcript variant 1, mRNA [NM_000803]	NM_000803
A_19_P00315824	-8.496442	down	4.84E-02	4.742E-03	9.754044	6.667185	6.7312202	PCAT19	Homo sapiens prostate cancer associated transcript 19 (non-protein coding) (PCAT19), long non-coding RNA [NR_040109]	NR_040109
A_23_P423309	-9.667998	down	4.41E-02	3.884E-03	5.121935	1.8487177	2.5800195	PCDH12	Homo sapiens protocadherin 12 (PCDH12), mRNA [NM_016580]	NM_016580
A_23_P76538	-10.971784	down	2.39E-02	1.404E-03	8.746446	5.2907195	3.4690785	TESC	Homo sapiens tescalcin (TESC), transcript variant 1, mRNA [NM_017899]	NM_017899
A_23_P29005	-8.046399	down	4.16E-02	3.567E-03	5.0134416	2.0050983	1.8488333	SAMSN1	Homo sapiens SAM domain, SH3 domain and nuclear localization signals 1 (SAMSN1), transcript variant 1, mRNA [NM_022136]	NM_022136
A_24_P209455	-56.957382	down	1.12E-03	3.819E-06	7.881413	2.0496023	1.9635624	GIMAP4	Homo sapiens GTPase, IMAP family member 4 (GIMAP4), mRNA [NM_018326]	NM_018326

A_23_P137931	-6.0947237	down	4.84E-02	4.703E-03	4.413346	1.805785	1.7906271	ADORA3	Homo sapiens adenosine A3 receptor (ADORA3), transcript variant A, mRNA [NM_000677]	NM_000677
A_23_P56328	-25.20382	down	1.62E-02	6.677E-04	8.544329	3.8887582	3.2549138	PLVAP	plasmalemma vesicle associated protein [Source:HGNC Symbol;Acc:HGNC:13635] [ENST00000252590]	AK096030
A_23_P19650	-4.191767	down	2.26E-02	1.172E-03	3.797865	1.7303064	1.7227244	VIP	Homo sapiens vasoactive intestinal peptide (VIP), transcript variant 1, mRNA [NM_003381]	NM_003381
A_23_P17481	-9.178282	down	2.78E-02	1.788E-03	5.188873	1.9906487	2.0975652	SIGLEC1	Homo sapiens sialic acid binding Ig-like lectin 1, sialoadhesin (SIGLEC1), mRNA [NM_023068]	NM_023068
A_24_P943301	-4.525474	down	4.57E-02	4.281E-03	4.9588227	2.7807539	2.7690248	PEAR1	Homo sapiens platelet endothelial aggregation receptor 1 (PEAR1), mRNA [NM_001080471]	NM_001080471
A_33_P3347452	-11.474197	down	2.02E-02	9.708E-04	8.077066	4.556745	3.3713844	RPS6KA2	Homo sapiens ribosomal protein S6 kinase, 90kDa, polypeptide 2 (RPS6KA2), transcript variant 1, mRNA [NM_021135]	NM_021135
A_23_P3643	-3.3867774	down	4.84E-02	4.736E-03	3.8007047	2.0407915	3.1672273	DNASE1L2	Homo sapiens deoxyribonuclease I-like 2 (DNASE1L2), transcript variant 1, mRNA [NM_001374]	NM_001374
A_33_P3415430	-4.5098205	down	3.49E-02	2.743E-03	12.360315	10.187245	10.90069	HSPA1B	Homo sapiens heat shock 70kDa protein 1B (HSPA1B), mRNA [NM_005346]	NM_005346
A_23_P142447	-6.816549	down	3.98E-03	6.703E-05	8.379015	5.6099734	5.663677	MYO1F	Homo sapiens myosin IF (MYO1F), mRNA [NM_012335]	NM_012335
A_23_P217428	-5.183864	down	2.31E-02	1.221E-03	4.1952915	1.8212636	1.7804794	ARHGAP6	Homo sapiens Rho GTPase activating protein 6 (ARHGAP6), transcript variant 1, mRNA [NM_013427]	NM_013427
A_23_P8108	-8.737076	down	2.33E-02	1.327E-03	9.132545	6.005395	5.023675	HLA-DQB1	Homo sapiens major histocompatibility complex, class II, DQ beta 1 (HLA-DQB1), transcript variant 3, mRNA [NM_001243962]	NM_001243962
A_22_P00001149	-2.2823474	down	2.33E-02	1.323E-03	6.5212007	5.3306823	5.3748198	PCED1B-AS1	PCED1B antisense RNA 1 [Source:HGNC Symbol;Acc:HGNC:44166] [ENST00000547851]	AK092995
A_32_P351968	-15.454137	down	3.15E-02	2.249E-03	9.470667	5.5207458	3.0833707	HLA-DMB	Homo sapiens major histocompatibility complex, class II, DM beta (HLA-DMB), mRNA [NM_002118]	NM_002118
A_21_P0000024	-35.067112	down	1.28E-02	4.344E-04	8.164593	3.032546	2.332002	FLT1	Homo sapiens fms-related tyrosine kinase 1 (FLT1), transcript variant 4, mRNA [NM_001160031]	NM_001160031
A_23_P45099	-25.553152	down	1.79E-03	1.829E-05	10.294619	5.6191893	1.7380695	HLA-DRB5	Homo sapiens major histocompatibility complex, class II, DR beta 5 (HLA-DRB5), mRNA [NM_002125]	NM_002125
A_23_P34644	-8.816733	down	2.32E-02	1.291E-03	5.6735415	2.5332973	1.9789033	FCGR2B	Homo sapiens Fc fragment of IgG, low affinity IIb, receptor (CD32) (FCGR2B), transcript variant 1, mRNA [NM_004001]	NM_004001
A_33_P3395314	-22.352425	down	3.65E-03	5.685E-05	6.5783424	2.095983	3.2254772			
A_33_P3281695	-3.922371	down	1.18E-02	3.610E-04	4.297182	2.3254561	1.8864207	NLRP3	Homo sapiens NLR family, pyrin domain containing 3 (NLRP3), transcript variant 6, mRNA [NM_001243133]	NM_001243133
A_22_P00003850	-6.968093	down	2.62E-02	1.651E-03	6.1644697	3.3637059	2.0599988	LINC01272	Homo sapiens long intergenic non-protein coding RNA 1272 (LINC01272), mRNA [NM_001278655]	NM_001278655
A_23_P42746	-8.417564	down	3.61E-02	2.883E-03	5.23482	2.161417	2.412321	NCF1	Homo sapiens neutrophil cytosolic factor 1 (NCF1), mRNA [NM_000265]	NM_000265
A_33_P3354604	-6.731958	down	2.33E-02	1.308E-03	5.254097	2.5030708	2.548044	CCL4L2	Homo sapiens chemokine (C-C motif) ligand 4-like 2 (CCL4L2), transcript variant CCL4L2b2, mRNA [NM_001291470]	NM_001291470
A_23_P253350	-9.247047	down	2.35E-02	1.353E-03	5.300724	2.0917313	2.44296	C8orf4	Homo sapiens chromosome 8 open reading frame 4 (C8orf4), mRNA [NM_020130]	NM_020130
A_23_P114903	-9.110917	down	5.62E-03	1.147E-04	7.7671804	4.579584	5.0904765	HSPA6	Homo sapiens heat shock 70kDa protein 6 (HSP70B') (HSPA6), mRNA [NM_002155]	NM_002155
A_23_P39386	-5.7741847	down	9.28E-03	2.461E-04	10.07419	7.544573	7.324834	HCST	Homo sapiens hematopoietic cell signal transducer (HCST), transcript variant 1, mRNA [NM_014266]	NM_014266
A_23_P214627	-57.58914	down	2.27E-02	1.182E-03	11.226027	5.3783016	1.7963964	AIF1	Homo sapiens allograft inflammatory factor 1 (AIF1), transcript variant 2, mRNA [NM_004847]	NM_004847
A_21_P0005146	-3.071683	down	4.41E-02	3.903E-03	3.6513722	2.032343	2.23365	lnc-FAM120B-6	LNCipedia lincRNA (lnc-FAM120B-6), lincRNA [lnc-FAM120B-6:1]	
A_24_P381505	-2.1656651	down	1.82E-02	8.290E-04	4.7387333	3.623923	2.8821268	GLIPR1L2	Homo sapiens GLI pathogenesis-related 1 like 2 (GLIPR1L2), transcript variant 2, mRNA [NM_152436]	NM_152436
A_33_P3260614	-4.5588655	down	4.92E-02	4.928E-03	8.758802	6.5701275	8.711955	PLCB2	Homo sapiens phospholipase C, beta 2 (PLCB2), transcript variant 1, mRNA [NM_004573]	NM_004573
A_23_P145096	-26.954206	down	2.47E-02	1.487E-03	6.7878947	2.0354564	1.7366457	PLA2G7	Homo sapiens phospholipase A2, group VII (platelet-activating factor acetylhydrolase, plasma) (PLA2G7), transcript variant 1, mRNA [NM_005084]	NM_005084
A_22_P00021967	-6.235055	down	2.74E-02	1.749E-03	5.112222	2.4718199	3.0393438			
A_22_P00002572	-4.3804617	down	2.56E-02	1.580E-03	4.127658	1.9965748	1.9118009	LOC100506388	Homo sapiens uncharacterized LOC100506388 (LOC100506388), transcript variant 1, mRNA [NM_001242780]	NM_001242780
A_33_P3260654	-38.56518	down	2.32E-02	1.285E-03	7.473015	2.2037876	2.8726213		T cell receptor beta constant 1 [Source:HGNC Symbol;Acc:HGNC:12156] [ENST00000610439]	EU030678
A_23_P357284	-17.115877	down	3.80E-02	3.078E-03	8.002087	3.904823	2.5731583	GPR4	Homo sapiens G protein-coupled receptor 4 (GPR4), mRNA [NM_005282]	NM_005282
A_33_P3262635	-31.43896	down	1.74E-03	1.665E-05	7.3907447	2.416263	2.6226783	CECR1	Homo sapiens cat eye syndrome chromosome region, candidate 1 (CECR1), transcript variant 3, mRNA [NM_001282225]	NM_001282225
A_33_P3224710	-9.460724	down	1.97E-03	2.192E-05	4.96912	1.7271695	1.7166363	TFEC	Homo sapiens transcription factor EC (TFEC), transcript variant 1, mRNA [NM_012252]	NM_012252
A_33_P3267799	-10.352207	down	3.31E-02	2.471E-03	5.894442	2.5225756	1.7456462	LILRB4	Homo sapiens leukocyte immunoglobulin-like receptor, subfamily B (with TM and ITIM domains), member 4 (LILRB4), transcript variant 1, mRNA [NM_001278426]	NM_001278426
A_23_P49816	-10.598544	down	1.00E-02	2.839E-04	10.325027	6.9192333	3.197973	ADAP2	Homo sapiens ArfGAP with dual PH domains 2 (ADAP2), mRNA [NM_018404]	NM_018404
A_22_P00013663	-3.4355783	down	2.02E-02	9.892E-04	3.5067773	1.7262243	1.8835738	lnc-RP11-680F20.4.1-2	LNCipedia lincRNA (lnc-RP11-680F20.4.1-2), lincRNA [lnc-RP11-680F20.4.1-2:2]	
A_23_P4773	-42.05727	down	4.19E-03	7.476E-05	7.338848	1.944565	1.8311121	LILRB5	Homo sapiens leukocyte immunoglobulin-like receptor, subfamily B (with TM and ITIM domains), member 5 (LILRB5), transcript variant 2, mRNA [NM_006840]	NM_006840
A_33_P3370575	-11.434851	down	1.14E-03	4.456E-06	5.2325134	1.717148	1.7106786	CD200	Homo sapiens CD200 molecule (CD200), transcript variant 2, mRNA [NM_001004196]	NM_001004196
A_23_P130113	-5.0134974	down	9.70E-03	2.647E-04	4.559923	2.2341058	3.1676865	ASGR2	Homo sapiens asialoglycoprotein receptor 2 (ASGR2), transcript variant H2', mRNA [NM_080912]	NM_080912
A_23_P207564	-23.648846	down	9.21E-03	2.418E-04	8.672113	4.1084156	2.6719205	CCL4L2	Homo sapiens chemokine (C-C motif) ligand 4-like 2 (CCL4L2), transcript variant CCL4L2b2, mRNA [NM_001291470]	NM_001291470
A_33_P3421351	-11.692541	down	3.63E-02	2.909E-03	5.858863	2.3113463	5.1388836	TRAF3IP3	Homo sapiens TRAF3 interacting protein 3 (TRAF3IP3), transcript variant 2, mRNA [NM_001287754]	NM_001287754
A_22_P00014094	-51.379932	down	4.39E-04	7.057E-07	7.928477	2.2453434	1.8895507	SASH3	Homo sapiens SAM and SH3 domain containing 3 (SASH3), mRNA [NM_018990]	NM_018990
A_24_P232365	-10.003642	down	3.41E-02	2.633E-03	6.0244684	2.702015	2.218468	APBB1IP	Homo sapiens amyloid beta (A4) precursor protein-binding, family B, member 1 interacting protein (APBB1IP), mRNA [NM_019043]	NM_019043
A_24_P82749	-6.1848125	down	4.16E-02	3.558E-03	5.7402515	3.1115217	2.970683	CD37	Homo sapiens CD37 molecule (CD37), transcript variant 1, mRNA [NM_001774]	NM_001774
A_33_P3362611	-17.983524	down	3.38E-02	2.587E-03	7.0659137	2.89731	1.8910775	ELTD1	Homo sapiens EGF, latrophilin and seven transmembrane domain containing 1 (ELTD1), mRNA [NM_022159]	NM_022159
A_23_P314250	-4.002621	down	3.50E-03	5.092E-05	7.5346913	5.5337462	6.117605	FAM78A	Homo sapiens family with sequence similarity 78, member A (FAM78A), mRNA [NM_033387]	NM_033387
A_23_P13493	-6.813105	down	3.79E-02	3.063E-03	4.4986906	1.7303782	1.7190976	OR51E1	Homo sapiens olfactory receptor, family 51, subfamily E, member 1 (OR51E1), mRNA [NM_152430]	NM_152430
A_23_P12554	-3.4135804	down	4.47E-02	4.069E-03	3.5554242	1.7841384	1.7701377	PKD2L1	Homo sapiens polycystic kidney disease 2-like 1 (PKD2L1), transcript variant 1, mRNA [NM_016112]	NM_016112
A_24_P159434	-5.6398897	down	4.63E-03	8.495E-05	4.294293	1.7986261	1.7847524	CD300A	Homo sapiens CD300a molecule (CD300A), transcript variant 1, mRNA [NM_007261]	NM_007261

A_22_P00007720	-20.176544	down	1.74E-03	1.726E-05	10.115517	5.7809095	1.8261281	HLA-DQB1	Homo sapiens major histocompatibility complex, class II, DQ beta 1 (HLA-DQB1), transcript variant 2, mRNA [NM_001243961]	NM_001243961
A_23_P218770	-4.809393	down	3.15E-02	2.257E-03	9.273399	7.0075445	6.243514	RAC2	Homo sapiens ras-related C3 botulinum toxin substrate 2 (rho family, small GTP binding protein Rac2) (RAC2), mRNA [NM_002872]	NM_002872
A_23_P74547	-31.531076	down	2.22E-03	2.550E-05	7.301684	2.3229814	1.937281	CD53	Homo sapiens CD53 molecule (CD53), transcript variant 1, mRNA [NM_001040033]	NM_001040033
A_23_P74778	-3.8021564	down	3.28E-02	2.442E-03	12.22337	10.296552	11.198404	C1orf54	Homo sapiens chromosome 1 open reading frame 54 (C1orf54), transcript variant 3, mRNA [NM_024579]	NM_024579
A_23_P171074	-18.39085	down	1.69E-02	7.409E-04	7.446327	3.2454112	6.379173	ITM2A	Homo sapiens integral membrane protein 2A (ITM2A), transcript variant 1, mRNA [NM_004867]	NM_004867
A_23_P169257	-3.5178084	down	3.38E-02	2.579E-03	3.5488849	1.734208	1.7227188	TNFSF8	Homo sapiens tumor necrosis factor (ligand) superfamily, member 8 (TNFSF8), transcript variant 1, mRNA [NM_001244]	NM_001244
A_24_P165423	-13.778615	down	4.00E-02	3.359E-03	7.977746	4.193387	3.2356305	RBP7	Homo sapiens retinol binding protein 7, cellular (RBP7), mRNA [NM_052960]	NM_052960
A_23_P407565	-14.735864	down	1.00E-02	2.841E-04	6.020051	2.1387913	2.4984813	CX3CR1	Homo sapiens chemokine (C-X3-C motif) receptor 1 (CX3CR1), transcript variant 4, mRNA [NM_001337]	NM_001337
A_33_P3309526	-17.943033	down	9.69E-03	2.593E-04	6.2156324	2.0502806	2.350549	PTPRC	Homo sapiens protein tyrosine phosphatase, receptor type, C (PTPRC), transcript variant 1, mRNA [NM_002838]	NM_002838
A_22_P00004088	-11.106792	down	2.90E-02	1.924E-03	6.4615235	2.9881532	2.772223			AF086288
A_23_P28434	-10.960993	down	6.81E-03	1.544E-04	10.988644	7.534337	6.4377174	VAMP8	Homo sapiens vesicle-associated membrane protein 8 (VAMP8), mRNA [NM_003761]	NM_003761
A_23_P160849	-3.9476922	down	4.12E-02	3.496E-03	9.022164	7.041155	7.232574	FCER1G	Homo sapiens Fc fragment of IgE, high affinity I, receptor for; gamma polypeptide (FCER1G), mRNA [NM_004106]	NM_004106
A_23_P153524	-2.2030053	down	3.90E-02	3.196E-03	5.8121614	4.6726885	6.123244	C19orf73	Homo sapiens chromosome 19 open reading frame 73 (C19orf73), mRNA [NM_018111]	NM_018111
A_24_P148717	-42.197342	down	4.50E-04	1.146E-06	7.2481885	1.8491081	2.3373945	CCR1	Homo sapiens chemokine (C-C motif) receptor 1 (CCR1), mRNA [NM_001295]	NM_001295
A_23_P162386	-5.8293486	down	1.21E-02	3.888E-04	4.6184764	2.0751417	1.7620293	BIN2	Homo sapiens bridging integrator 2 (BIN2), transcript variant 1, mRNA [NM_016293]	NM_016293
A_23_P141429	-6.533615	down	4.99E-02	5.047E-03	7.3827133	4.674832	4.7960253	ABI3	Homo sapiens ABI family, member 3 (ABI3), transcript variant 1, mRNA [NM_016428]	NM_016428
A_19_P00320718	-29.676474	down	1.80E-02	8.178E-04	7.89283	3.001582	2.4306285	PCAT19	Homo sapiens prostate cancer associated transcript 19 (non-protein coding) (PCAT19), long non-coding RNA [NR_040109]	NR_040109
A_33_P3405213	-36.59028	down	4.53E-02	4.194E-03	8.499485	3.3060966	2.5801327	PECAM1	Homo sapiens platelet/endothelial cell adhesion molecule 1 (PECAM1), mRNA [NM_000442]	NM_000442
A_24_P192914	-97.29555	down	1.23E-03	5.340E-06	9.041411	2.4371095	2.4438047	AMICA1	Homo sapiens adhesion molecule, interacts with CXADR antigen 1 (AMICA1), transcript variant 2, mRNA [NM_153206]	NM_153206
A_33_P3222233	-2.6113489	down	1.36E-02	4.934E-04	3.0992913	1.7144961	2.523618	CC2D2B	Homo sapiens coiled-coil and C2 domain containing 2B (CC2D2B), transcript variant 1, mRNA [NM_001159747]	NM_001159747
A_23_P401700	-27.467697	down	4.47E-02	4.072E-03	7.7231545	2.9434905	1.895978	APBB1IP	Homo sapiens amyloid beta (A4) precursor protein-binding, family B, member 1 interacting protein (APBB1IP), mRNA [NM_019043]	NM_019043
A_23_P23279	-15.803401	down	1.87E-03	2.003E-05	5.6964517	1.7142885	1.714768	RCSD1	Homo sapiens RCSD domain containing 1 (RCSD1), mRNA [NM_052862]	NM_052862
A_23_P27795	-12.19999	down	1.28E-02	4.427E-04	8.092021	4.483213	3.7235768	SPINT2	Homo sapiens serine peptidase inhibitor, Kunitz type, 2 (SPINT2), transcript variant a, mRNA [NM_021102]	NM_021102
A_33_P3259203	-45.398624	down	1.46E-02	5.604E-04	8.761564	3.2569876	5.2463694	TMEM255B	transmembrane protein 255B [Source:HGNC Symbol;Acc:HGNC:28297] [ENST00000375353]	XM_006719983
A_24_P393740	-13.053086	down	1.18E-02	3.596E-04	5.563361	1.8570421	1.8972706	FYB	Homo sapiens FYN binding protein (FYB), transcript variant 1, mRNA [NM_001465]	NM_001465
A_24_P54390	- 15.6123295	down	2.34E-02	1.345E-03	6.1724224	2.2078085	1.8956738	RASGRP3	Homo sapiens RAS guanyl releasing protein 3 (calcium and DAG-regulated) (RASGRP3), transcript variant 2, mRNA [NM_170672]	NM_170672
A_23_P63390	-36.571632	down	2.89E-02	1.895E-03	8.26232	3.0696664	2.5077763	FCGR1B	Homo sapiens Fc fragment of IgG, high affinity Ib, receptor (CD64) (FCGR1B), transcript variant 1, mRNA [NM_001017986]	NM_001017986
A_23_P70688	-4.268147	down	2.15E-02	1.065E-03	8.744747	6.6511374	6.290273	LY86	Homo sapiens lymphocyte antigen 86 (LY86), mRNA [NM_004271]	NM_004271
A_19_P00808044	-55.488594	down	7.85E-03	1.980E-04	7.747858	1.9537386	1.8191707	PCAT19	Homo sapiens prostate cancer associated transcript 19 (non-protein coding) (PCAT19), long non-coding RNA [NR_040109]	NR_040109
A_23_P217901	-7.816332	down	2.47E-03	3.276E-05	5.031646	2.065154	1.9307294	TSTD1	Homo sapiens thiosulfate sulfurtransferase (rhodanese)-like domain containing 1 (TSTD1), transcript variant 1, mRNA [NM_001113207]	NM_001113207
A_33_P3281273	-14.574219	down	2.19E-02	1.116E-03	7.1828065	3.3174598	2.2425718	S1PR4	Homo sapiens sphingosine-1-phosphate receptor 4 (S1PR4), mRNA [NM_003775]	NM_003775
A_21_P0014497	-8.487237	down	4.78E-02	4.609E-03	4.8362713	1.7509763	1.7410145	LOC101928612	PREDICTED: Homo sapiens uncharacterized LOC101928612 (LOC101928612), transcript variant X1, ncRNA [XR_245345]	XR_245345
A_23_P98565	-11.989586	down	9.75E-03	2.686E-04	5.340844	1.7571342	2.8694618	MS4A14	Homo sapiens membrane-spanning 4-domains, subfamily A, member 14 (MS4A14), transcript variant 1, mRNA [NM_032597]	NM_032597
A_23_P103104	-18.28997	down	7.85E-03	1.981E-04	6.364608	2.1716273	1.719123	MFNG	Homo sapiens MFNG O-fucosylpeptide 3-beta-N-acetylglucosaminyltransferase (MFNG), transcript variant 1, mRNA [NM_002405]	NM_002405
A_23_P208493	-31.601334	down	1.28E-03	6.403E-06	6.886791	1.9048779	1.8533506	LILRB2	Homo sapiens leukocyte immunoglobulin-like receptor, subfamily B (with TM and ITIM domains), member 2 (LILRB2), transcript variant 1, mRNA [NM_005874]	NM_005874
A_23_P201778	-4.91427	down	3.80E-02	3.090E-03	4.1063223	1.8093451	2.2883413	PTPN7	Homo sapiens protein tyrosine phosphatase, non-receptor type 7 (PTPN7), transcript variant 2, mRNA [NM_080588]	NM_080588
A_23_P147711	-13.277663	down	1.28E-02	4.448E-04	5.8459935	2.1150641	2.8628836	NPR1	Homo sapiens natriuretic peptide receptor 1 (NPR1), mRNA [NM_000906]	NM_000906
A_24_P71973	-33.459232	down	1.74E-03	1.452E-05	7.1970153	2.1326828	1.9697335	KDR	Homo sapiens kinase insert domain receptor (a type III receptor tyrosine kinase) (KDR), mRNA [NM_002253]	NM_002253
A_23_P142075	-22.159286	down	6.24E-03	1.368E-04	10.747187	6.277347	5.038362	ACP5	Homo sapiens acid phosphatase 5, tartrate resistant (ACP5), transcript variant 4, mRNA [NM_001611]	NM_001611
A_23_P25155	-4.482456	down	1.75E-02	7.753E-04	4.643339	2.4790497	3.2453063	GPR84	Homo sapiens G protein-coupled receptor 84 (GPR84), mRNA [NM_020370]	NM_020370
A_24_P353638	-9.509677	down	2.46E-02	1.470E-03	5.5454903	2.296094	2.7051888	SLAMF7	Homo sapiens SLAM family member 7 (SLAMF7), transcript variant 1, mRNA [NM_021181]	NM_021181
A_24_P222655	-42.60068	down	2.43E-02	1.442E-03	9.269215	3.85641	1.7250166	C1QA	Homo sapiens complement component 1, q subcomponent, A chain (C1QA), mRNA [NM_015991]	NM_015991
A_23_P78608	-8.991813	down	5.96E-03	1.261E-04	5.556978	2.3883662	3.6445556	DENND1C	Homo sapiens DENN/MADD domain containing 1C (DENND1C), transcript variant 1, mRNA [NM_024898]	NM_024898
A_23_P155057	-31.202654	down	2.51E-02	1.523E-03	8.455984	3.4923873	1.7995181	CYTH4	Homo sapiens cytohesin 4 (CYTH4), mRNA [NM_013385]	NM_013385
A_23_P30913	-25.578663	down	1.38E-02	5.094E-04	11.487638	6.8107696	1.9952788	HLA-DPA1	Homo sapiens major histocompatibility complex, class II, DP alpha 1 (HLA-DPA1), transcript variant 1, mRNA [NM_033554]	NM_033554
A_33_P3293918	-11.797264	down	7.35E-03	1.724E-04	9.153873	5.593493	4.4692984	SH2D3C	Homo sapiens SH2 domain containing 3C (SH2D3C), transcript variant 1, mRNA [NM_170600]	NM_170600
A_23_P64661	-33.935055	down	2.39E-03	2.920E-05	7.56378	2.4790757	1.9108412	ARHGAP9	Homo sapiens Rho GTPase activating protein 9 (ARHGAP9), transcript variant 1, mRNA [NM_032496]	NM_032496
A_32_P56001	-31.595047	down	1.97E-02	9.362E-04	7.4194555	2.437829	2.396825	CD93	Homo sapiens CD93 molecule (CD93), mRNA [NM_012072]	NM_012072
A_23_P143621	-2.793077	down	4.47E-02	4.041E-03	3.242589	1.7607336	3.6628227	CRYBB1	Homo sapiens crystallin, beta B1 (CRYBB1), mRNA [NM_001887]	NM_001887
A_24_P64167	-3.7395594	down	2.90E-02	1.921E-03	4.360846	2.4579778	2.0863369	PTGS1	Homo sapiens prostaglandin-endoperoxide synthase 1 (prostaglandin G/H synthase and cyclooxygenase) (PTGS1), transcript variant 1, mRNA [NM_000962]	NM_000962

A_33_P3332970	-4.6972375	down	3.20E-02	2.353E-03	5.9509516	3.719139	3.738103	CLEC2B	Homo sapiens C-type lectin domain family 2, member B (CLEC2B), mRNA [NM_005127]	NM_005127
A_24_P295010	-10.462926	down	4.11E-04	3.145E-07	5.1060286	1.7188141	1.7238295	SERPINB9	Homo sapiens serpin peptidase inhibitor, clade B (ovalbumin), member 9 (SERPINB9), mRNA [NM_004155]	NM_004155
A_24_P132383	-13.827485	down	4.13E-03	7.268E-05	5.9173336	2.1278667	1.8754807	GIMAP8	Homo sapiens GTPase, IMAP family member 8 (GIMAP8), mRNA [NM_175571]	NM_175571
A_33_P3363637	-10.612658	down	1.57E-03	1.002E-05	5.147038	1.7393239	2.4961314	BLNK	Homo sapiens B-cell linker (BLNK), transcript variant 1, mRNA [NM_013314]	NM_013314
A_23_P119353	-17.052927	down	3.20E-02	2.365E-03	10.288305	6.1963577	7.4467077	RASIP1	Homo sapiens Ras interacting protein 1 (RASIP1), mRNA [NM_017805]	NM_017805
A_23_P104464	-34.527203	down	1.46E-02	5.524E-04	7.1164274	2.0067658	1.8905094	ALOX5	Homo sapiens arachidonate 5-lipoxygenase (ALOX5), transcript variant 1, mRNA [NM_000698]	NM_000698
A_23_P19510	-7.546725	down	2.31E-02	1.232E-03	6.921971	4.00612	3.8763793	HLA-DQB2	Homo sapiens major histocompatibility complex, class II, DQ beta 2 (HLA-DQB2), transcript variant 2, mRNA [NM_001198858]	NM_001198858
A_23_P152428	-5.028871	down	5.09E-03	9.986E-05	4.298614	1.9683794	2.481165	MARVELD3	Homo sapiens MARVEL domain containing 3 (MARVELD3), transcript variant 2, mRNA [NM_052858]	NM_052858
A_23_P42588	-15.888238	down	1.57E-03	9.279E-06	5.8523107	1.8624233	1.8191569	GIMAP5	Homo sapiens GTPase, IMAP family member 5 (GIMAP5), mRNA [NM_018384]	NM_018384
A_24_P274831	-96.41034	down	6.81E-05	1.737E-08	8.336134	1.745018	1.9654166	GIMAP7	Homo sapiens GTPase, IMAP family member 7 (GIMAP7), mRNA [NM_153236]	NM_153236
A_24_P376391	-2.28487	down	1.66E-02	7.200E-04	11.211205	10.019093	10.160099	PLXND1	Homo sapiens plexin D1 (PLXND1), mRNA [NM_015103]	NM_015103
A_23_P68121	-3.2902646	down	3.15E-02	2.261E-03	3.5402412	1.8220376	3.381834	PSD4	Homo sapiens pleckstrin and Sec7 domain containing 4 (PSD4), mRNA [NM_012455]	NM_012455
A_32_P87697	-30.917019	down	4.50E-02	4.131E-03	9.463553	4.513224	1.7267615	HLA-DRA	Homo sapiens major histocompatibility complex, class II, DR alpha (HLA-DRA), mRNA [NM_019111]	NM_019111
A_23_P218369	-21.500725	down	3.09E-02	2.145E-03	7.516018	3.0897043	3.4217427	CCL14	Homo sapiens chemokine (C-C motif) ligand 14 (CCL14), transcript variant 3, mRNA [NM_032963]	NM_032963
A_23_P79069	-6.513126	down	2.32E-02	1.285E-03	9.629028	6.9256783	6.243986	RASAL3	Homo sapiens RAS protein activator like 3 (RASAL3), mRNA [NM_022904]	NM_022904
A_23_P83098	-8.115382	down	2.62E-02	1.652E-03	5.5987844	2.5781255	2.0708776	ALDH1A1	Homo sapiens aldehyde dehydrogenase 1 family, member A1 (ALDH1A1), mRNA [NM_000689]	NM_000689
A_33_P3364821	-34.592438	down	1.65E-02	7.081E-04	8.618453	3.5060685	2.6705446	PTPRC	Homo sapiens protein tyrosine phosphatase, receptor type, C (PTPRC), transcript variant 1, mRNA [NM_002838]	NM_002838
A_23_P75769	-35.01143	down	1.21E-02	3.835E-04	8.288646	3.1588917	3.3740919	MS4A4A	Homo sapiens membrane-spanning 4-domains, subfamily A, member 4A (MS4A4A), transcript variant 2, mRNA [NM_024021]	NM_024021
A_23_P382065	-51.88793	down	3.59E-03	5.345E-05	7.500296	1.802969	2.516707	EMCN	Homo sapiens endomucin (EMCN), transcript variant 1, mRNA [NM_016242]	NM_016242
A_23_P212655	-7.3689327	down	2.90E-02	1.958E-03	5.146054	2.2645981	1.9197505	KLHL6	Homo sapiens kelch-like family member 6 (KLHL6), mRNA [NM_130446]	NM_130446
A_23_P12746	-16.732304	down	1.92E-02	8.890E-04	5.914506	1.8499416	1.8050408	MRC1	Homo sapiens mannose receptor, C type 1 (MRC1), mRNA [NM_002438]	NM_002438
A_23_P117662	-12.766525	down	4.52E-02	4.171E-03	7.1616917	3.4873977	4.196031	HDC	Homo sapiens histidine decarboxylase (HDC), mRNA [NM_002112]	NM_002112
A_33_P3343120	-22.77904	down	1.74E-03	1.493E-05	6.321413	1.811778	2.0350127	IRF8	Homo sapiens interferon regulatory factor 8 (IRF8), mRNA [NM_002163]	NM_002163
A_23_P150768	-27.56982	down	2.16E-02	1.077E-03	7.8200483	3.0350301	1.7319604	SLCO2B1	Homo sapiens solute carrier organic anion transporter family, member 2B1 (SLCO2B1), transcript variant 1, mRNA [NM_007256]	NM_007256
A_23_P72697	-9.351914	down	3.63E-02	2.914E-03	8.360227	5.134965	4.665314	GPIHBP1	Homo sapiens glycosylphosphatidylinositol anchored high density lipoprotein binding protein 1 (GPIHBP1), transcript variant 1, mRNA [NM_178172]	NM_178172
A_23_P430411	-2.2640977	down	4.92E-02	4.936E-03	3.5698285	2.3908923	4.004017	ITGB2	integrin, beta 2 (complement component 3 receptor 3 and 4 subunit) [Source:HGNC Symbol;Acc:HGNC:6155] [ENST00000610622]	L78790
A_33_P3842556	-27.61616	down	5.09E-03	9.734E-05	7.668912	2.8814712	3.1743855	IKZF1	Homo sapiens IKAROS family zinc finger 1 (Ikaros) (IKZF1), transcript variant 1, mRNA [NM_006060]	NM_006060
A_23_P312132	-40.147484	down	1.12E-03	4.013E-06	7.370801	2.0435634	1.7783923	ITGAX	Homo sapiens integrin, alpha X (complement component 3 receptor 4 subunit) (ITGAX), transcript variant 2, mRNA [NM_000887]	NM_000887
A_32_P217750	-14.0173025	down	1.65E-02	7.121E-04	8.067239	4.258102	3.9113636	IL3RA	Homo sapiens interleukin 3 receptor, alpha (low affinity) (IL3RA), transcript variant 1, mRNA [NM_002183]	NM_002183
A_23_P119478	-6.3145294	down	4.47E-02	4.094E-03	4.439541	1.7808656	1.7812424	EBI3	Homo sapiens Epstein-Barr virus induced 3 (EBI3), mRNA [NM_005755]	NM_005755
A_23_P15414	-4.244938	down	4.16E-02	3.550E-03	7.456785	5.371042	4.8293695	SCARF1	Homo sapiens scavenger receptor class F, member 1 (SCARF1), transcript variant 1, mRNA [NM_003693]	NM_003693
A_33_P3358731	-3.0977051	down	2.42E-02	1.428E-03	6.571018	4.9398184	6.6458206	PCSK5	Homo sapiens proprotein convertase subtilisin/kexin type 5 (PCSK5), transcript variant 2, mRNA [NM_006200]	NM_006200
A_33_P3281403	-5.679054	down	3.41E-02	2.625E-03	5.518863	3.0132127	4.7143154	TRAF3IP3	Homo sapiens TRAF3 interacting protein 3 (TRAF3IP3), transcript variant 2, mRNA [NM_001287754]	NM_001287754
A_33_P3314794	-7.1901083	down	1.62E-02	6.713E-04	5.013661	2.1676474	3.274991	IQCA1	Homo sapiens IQ motif containing with AAA domain 1 (IQCA1), transcript variant 1, mRNA [NM_024726]	NM_024726
A_23_P215060	-24.744646	down	1.01E-02	2.892E-04	11.32992	6.7008753	4.987583	PODXL	Homo sapiens podocalyxin-like (PODXL), transcript variant 1, mRNA [NM_001018111]	NM_001018111
A_23_P70095	-26.286688	down	5.09E-03	9.782E-05	7.1672583	2.4509978	2.1906507	CD74	Homo sapiens CD74 molecule, major histocompatibility complex, class II invariant chain (CD74), transcript variant 3, mRNA [NM_001025158]	NM_001025158
A_23_P9883	-14.832426	down	1.97E-03	2.205E-05	6.1986713	2.3079886	2.2638693	NLRP3	Homo sapiens NLR family, pyrin domain containing 3 (NLRP3), transcript variant 3, mRNA [NM_001079821]	NM_001079821
A_23_P134914	-8.074149	down	2.59E-02	1.614E-03	4.8568254	1.8435152	1.822475	LY6H	Homo sapiens lymphocyte antigen 6 complex, locus H (LY6H), transcript variant 1, mRNA [NM_002347]	NM_002347
A_33_P3232688	-7.2146683	down	1.23E-02	4.049E-04	5.2648096	2.4138765	2.5577228		colony stimulating factor 2 receptor, alpha, low-affinity (granulocyte-macrophage) [Source:HGNC Symbol;Acc:HGNC:2435] [ENST00000381524]	
A_24_P102821	-7.307514	down	2.69E-03	3.766E-05	4.6914577	1.822077	1.8120251	PTAFR	Homo sapiens platelet-activating factor receptor (PTAFR), transcript variant 3, mRNA [NM_000952]	NM_000952
A_33_P3234277	-11.525053	down	2.02E-02	9.618E-04	9.672954	6.146252	5.639378	HLA-DPA1	Homo sapiens major histocompatibility complex, class II, DP alpha 1 (HLA-DPA1), transcript variant 2, mRNA [NM_001242524]	NM_001242524
A_33_P3397599	-6.8288445	down	1.28E-03	6.631E-06	4.509812	1.7381704	2.956911	LILRA6	Homo sapiens leukocyte immunoglobulin-like receptor, subfamily A (with TM domain), member 6 (LILRA6), transcript variant 2, non-coding RNA [NR_104098]	NR_104098
A_23_P501713	-5.9639463	down	4.56E-02	4.245E-03	5.2162457	2.6399784	3.853999	IL1F10	Homo sapiens interleukin 1 family, member 10 (theta) (IL1F10), transcript variant 1, mRNA [NM_032556]	NM_032556
A_23_P429977	-23.398151	down	2.25E-02	1.152E-03	8.035948	3.487625	2.3431072	KCNQ1	Homo sapiens potassium voltage-gated channel, KQT-like subfamily, member 1 (KCNQ1), transcript variant 1, mRNA [NM_000218]	NM_000218
A_33_P3241269	-2.9620051	down	9.70E-03	2.632E-04	3.7205534	2.1539793	1.9568843	CES1	Homo sapiens carboxylesterase 1 (CES1), transcript variant 1, mRNA [NM_001025195]	NM_001025195
A_33_P3293049	-38.979294	down	4.84E-02	4.719E-03	8.894259	3.6096234	1.8756276	HLA-DQA1	Homo sapiens major histocompatibility complex, class II, DQ alpha 1 (HLA-DQA1), mRNA [NM_002122]	NM_002122
A_32_P234184	-7.1145706	down	3.15E-02	2.263E-03	6.204022	3.3732452	1.766767	HES5	Homo sapiens hes family bHLH transcription factor 5 (HES5), mRNA [NM_001010926]	NM_001010926
A_24_P788878	-12.338916	down	2.02E-02	9.802E-04	6.349378	2.7242343	3.7801461	C2CD4B	Homo sapiens C2 calcium-dependent domain containing 4B (C2CD4B), mRNA [NM_001007595]	NM_001007595
A_23_P23829	-9.286817	down	4.35E-02	3.796E-03	5.393614	2.1784296	1.9927781	CD34	Homo sapiens CD34 molecule (CD34), transcript variant 2, mRNA [NM_001773]	NM_001773

A_23_P349966	-3.0819337	down	2.47E-02	1.487E-03	3.3622007	1.7383649	1.7383547	TMEM130	Homo sapiens transmembrane protein 130 (TMEM130), transcript variant 2, mRNA [NM_152913]	NM_152913
A_32_P210642	-12.861407	down	2.17E-02	1.096E-03	9.948479	6.263502	5.4457808	EGFL7	Homo sapiens EGF-like-domain, multiple 7 (EGFL7), transcript variant 2, mRNA [NM_201446]	NM_201446
A_24_P370472	-19.498404	down	3.09E-02	2.160E-03	8.735304	4.45002	2.6026716	HLA-DRB4	Homo sapiens major histocompatibility complex, class II, DR beta 4 (HLA-DRB4), mRNA [NM_021983]	NM_021983
A_23_P166087	-4.8301706	down	2.90E-02	1.965E-03	8.833349	6.561275	9.185312	RASSF2	Homo sapiens Ras association (RalGDS/AF-6) domain family member 2 (RASSF2), transcript variant 1, mRNA [NM_014737]	NM_014737
A_32_P85999	-46.091084	down	1.74E-03	1.532E-05	7.593442	2.067026	1.7403121	CDH13	Homo sapiens cadherin 13 (CDH13), transcript variant 1, mRNA [NM_001257]	NM_001257
A_23_P119835	-9.688362	down	1.35E-02	4.815E-04	5.583983	2.3077302	1.7394083	NLRC4	Homo sapiens NLR family, CARD domain containing 4 (NLRC4), transcript variant 1, mRNA [NM_021209]	NM_021209
A_23_P502336	-12.011689	down	6.78E-03	1.522E-04	5.5023365	1.9159694	2.3866901	EMR2	Homo sapiens egf-like module containing, mucin-like, hormone receptor-like 2 (EMR2), transcript variant 1, mRNA [NM_013447]	NM_013447
A_23_P1083	-8.044343	down	3.18E-02	2.313E-03	8.182267	5.1742926	2.8681803	GJA4	Homo sapiens gap junction protein, alpha 4, 37kDa (GJA4), mRNA [NM_002060]	NM_002060
A_23_P41390	-7.89289	down	3.38E-02	2.591E-03	5.5159616	2.535408	3.501287	SH3TC1	Homo sapiens SH3 domain and tetratricopeptide repeats 1 (SH3TC1), mRNA [NM_018986]	NM_018986
A_33_P3354256	-4.7519493	down	4.84E-02	4.754E-03	4.8484	2.5998807	2.333428	MTSS1	Homo sapiens metastasis suppressor 1 (MTSS1), transcript variant 1, mRNA [NM_001282971]	NM_001282971
A_22_P00017885	-3.120378	down	3.64E-02	2.930E-03	3.932935	2.2912142	2.6520448			
A_23_P54770	-10.531971	down	5.29E-03	1.065E-04	5.549832	2.1531284	1.9372051	APOBR	Homo sapiens apolipoprotein B receptor (APOBR), mRNA [NM_018690]	NM_018690
A_23_P501822	-4.755525	down	4.47E-02	4.030E-03	8.523161	6.273556	6.053876	JUP	Homo sapiens junction plakoglobin (JUP), transcript variant 1, mRNA [NM_002230]	NM_002230
A_23_P66694	-5.8736367	down	2.31E-02	1.224E-03	6.489062	3.9348078	4.192379	EVI2B	Homo sapiens ecotropic viral integration site 2B (EVI2B), mRNA [NM_006495]	NM_006495
A_24_P228130	-19.833874	down	1.28E-03	6.848E-06	6.742334	2.432439	1.938767	CCL3L3	Homo sapiens chemokine (C-C motif) ligand 3-like 3 (CCL3L3), mRNA [NM_001001437]	NM_001001437
A_23_P48596	-37.63382	down	4.41E-02	3.917E-03	12.964043	7.730085	5.537648	RNASE1	Homo sapiens ribonuclease, RNase A family, 1 (pancreatic) (RNASE1), transcript variant 3, mRNA [NM_198232]	NM_198232
A_23_P259621	-9.928419	down	1.28E-02	4.475E-04	9.076138	5.7645736	5.694812	LAT2	Homo sapiens linker for activation of T cells family, member 2 (LAT2), transcript variant 1, mRNA [NM_032464]	NM_032464
A_24_P103469	-7.5002766	down	1.69E-02	7.389E-04	7.7312236	4.82428	3.0848627	LST1	Homo sapiens leukocyte specific transcript 1 (LST1), transcript variant 1, mRNA [NM_007161]	NM_007161
A_33_P3209651	-4.4218097	down	2.33E-02	1.315E-03	3.8889143	1.7442775	2.0952373	WDFY4	Homo sapiens WDFY family member 4 (WDFY4), mRNA [NM_020945]	NM_020945
A_23_P209678	-6.365542	down	4.05E-03	7.026E-05	5.7863355	3.1160522	2.499489	PLEK	Homo sapiens pleckstrin (PLEK), mRNA [NM_002664]	NM_002664
A_33_P3231414	-13.882396	down	4.32E-03	7.814E-05	5.8720875	2.0769029	1.7683637	LILRB1	Homo sapiens leukocyte immunoglobulin-like receptor, subfamily B (with TM and ITIM domains), member 1 (LILRB1), transcript variant 1, mRNA [NM_006669]	NM_006669
A_33_P3328559	-11.608732	down	1.58E-02	6.261E-04	7.8397007	4.302562	5.686454	TBC1D10C	Homo sapiens TBC1 domain family, member 10C (TBC1D10C), transcript variant 1, mRNA [NM_198517]	NM_198517
A_33_P3354607	-39.116108	down	2.69E-03	3.753E-05	7.4850016	2.1953106	1.9167371	CCL4L2	Homo sapiens chemokine (C-C motif) ligand 4-like 2 (CCL4L2), transcript variant CCL4L2b2, mRNA [NM_001291470]	NM_001291470
A_32_P70158	-14.022238	down	2.74E-02	1.752E-03	8.505291	4.6956463	5.124997	LILRB3	Homo sapiens leukocyte immunoglobulin-like receptor, subfamily B (with TM and ITIM domains), member 3 (LILRB3), transcript variant 2, mRNA [NM_006864]	NM_006864
A_33_P3379268	-3.9848883	down	2.90E-02	1.928E-03	4.6254716	2.6309323	3.4561534	SCIMP	Homo sapiens SLP adaptor and CSK interacting membrane protein (SCIMP), transcript variant 1, mRNA [NM_207103]	NM_207103
A_23_P30547	-37.450264	down	1.57E-03	1.083E-05	7.519321	2.292417	1.7896895	LCP2	Homo sapiens lymphocyte cytosolic protein 2 (SH2 domain containing leukocyte protein of 76kDa) (LCP2), mRNA [NM_005565]	NM_005565
A_23_P255444	-4.7283983	down	4.32E-02	3.744E-03	4.2041306	1.9627789	2.3886375	DAPP1	Homo sapiens dual adaptor of phosphotyrosine and 3-phosphoinositides (DAPP1), mRNA [NM_014395]	NM_014395
A_32_P140139	-18.662628	down	4.33E-02	3.765E-03	9.263975	5.041895	1.9835631	F13A1	Homo sapiens coagulation factor XIII, A1 polypeptide (F13A1), mRNA [NM_000129]	NM_000129
A_23_P165136	-12.545743	down	1.74E-03	1.650E-05	5.8573413	2.2082152	2.7203705	LRRC25	Homo sapiens leucine rich repeat containing 25 (LRRC25), mRNA [NM_145256]	NM_145256
A_33_P3489646	-9.375611	down	1.62E-02	6.737E-04	8.059141	4.8302283	4.740722	SPI1	Homo sapiens Spi-1 proto-oncogene (SPI1), transcript variant 1, mRNA [NM_001080547]	NM_001080547
A_23_P46356	-13.673977	down	6.11E-03	1.309E-04	6.4773536	2.7039926	4.67439	TNFAIP8L2	Homo sapiens tumor necrosis factor, alpha-induced protein 8-like 2 (TNFAIP8L2), mRNA [NM_024575]	NM_024575
A_23_P167389	-9.836843	down	1.58E-02	6.242E-04	8.406333	5.1081376	5.047123	ARAP3	Homo sapiens ArfGAP with RhoGAP domain, ankyrin repeat and PH domain 3 (ARAP3), mRNA [NM_022481]	NM_022481
A_23_P103765	-9.054872	down	2.48E-03	3.352E-05	4.9561377	1.7774435	1.759114	FCER1A	Homo sapiens Fc fragment of IgE, high affinity I, receptor for; alpha polypeptide (FCER1A), mRNA [NM_002001]	NM_002001
A_23_P38795	-4.6965775	down	2.32E-02	1.266E-03	7.608864	5.377254	4.675023	FPR1	Homo sapiens formyl peptide receptor 1 (FPR1), transcript variant 2, mRNA [NM_002029]	NM_002029
A_23_P97141	-36.660744	down	1.21E-02	3.809E-04	7.30524	2.109076	2.2348874	RGS1	Homo sapiens regulator of G-protein signaling 1 (RGS1), mRNA [NM_002922]	NM_002922
A_33_P3271635	-12.753295	down	2.32E-02	1.252E-03	8.2282915	4.5554934	5.8125486	HLA-DPB1	Homo sapiens major histocompatibility complex, class II, DP beta 1 (HLA-DPB1), mRNA [NM_002121]	NM_002121
A_24_P116535	-6.0826826	down	1.30E-02	4.587E-04	8.046312	5.4416046	6.0412574	MMP15	Homo sapiens matrix metallopeptidase 15 (membrane-inserted) (MMP15), mRNA [NM_002428]	NM_002428
A_24_P202840	-5.321192	down	4.71E-02	4.476E-03	4.4370546	2.0253053	2.6319134	SIRPB2	Homo sapiens signal-regulatory protein beta 2 (SIRPB2), transcript variant 1, mRNA [NM_001122962]	NM_001122962
A_23_P136683	-33.3854	down	1.57E-03	1.059E-05	7.400058	2.3389125	1.8644826	HLA-DQB1	Homo sapiens major histocompatibility complex, class II, DQ beta 1 (HLA-DQB1), transcript variant 2, mRNA [NM_001243961]	NM_001243961
A_33_P3304170	-7.179095	down	2.16E-02	1.082E-03	5.584837	2.741035	2.6249373	PIK3CG	Homo sapiens phosphatidylinositol-4,5-bisphosphate 3-kinase, catalytic subunit gamma (PIK3CG), transcript variant 1, mRNA [NM_002649]	NM_002649
A_33_P3403576	-31.26261	down	2.80E-02	1.806E-03	8.9291115	3.9627452	4.22223	FCGR2C	Homo sapiens Fc fragment of IgG, low affinity IIc, receptor for (CD32) (gene/pseudogene) (FCGR2C), transcript variant 1, mRNA [NM_201563]	NM_201563
A_33_P3271651	-13.361881	down	1.80E-02	8.111E-04	10.023788	6.283737	4.7308073	HLA-DPB1	Homo sapiens major histocompatibility complex, class II, DP beta 1 (HLA-DPB1), mRNA [NM_002121]	NM_002121
A_23_P124108	-14.5503845	down	1.57E-03	1.003E-05	6.1358724	2.272887	3.9242659	ITGAM	Homo sapiens integrin, alpha M (complement component 3 receptor 3 subunit) (ITGAM), transcript variant 2, mRNA [NM_000632]	NM_000632
A_23_P85716	-16.89412	down	2.62E-02	1.649E-03	8.500572	4.422123	5.7144947	FCGR2A	Homo sapiens Fc fragment of IgG, low affinity IIa, receptor (CD32) (FCGR2A), transcript variant 2, mRNA [NM_021642]	NM_021642
A_23_P217269	-68.69325	down	2.32E-02	1.277E-03	9.602003	3.4999065	2.6209302	VSIG4	Homo sapiens V-set and immunoglobulin domain containing 4 (VSIG4), transcript variant 1, mRNA [NM_007268]	NM_007268
A_23_P72651	-22.19549	down	1.65E-02	7.068E-04	6.7847033	2.3125088	3.4307234	ECSCR	Homo sapiens endothelial cell surface expressed chemotaxis and apoptosis regulator (ECSCR), transcript variant 1, mRNA [NM_001077693]	NM_001077693
A_33_P3261953	-2.2363958	down	4.00E-02	3.352E-03	4.3604217	3.1992462	4.342726	LOC100996349	Homo sapiens testis expressed 264 pseudogene (LOC100996349), non-coding RNA [NR_103470]	NR_103470
A_33_P3215363	-3.871501	down	4.57E-02	4.286E-03	4.03573	2.0828369	4.9140463			
A_33_P3327617	-25.179182	down	1.63E-02	6.829E-04	7.286284	2.6321242	5.027398	FCGR2B	Homo sapiens Fc fragment of IgG, low affinity IIb, receptor (CD32) (FCGR2B), transcript variant 3, mRNA [NM_001002274]	NM_001002274

A_23_P47410	-7.110962	down	4.98E-02	5.018E-03	6.6533866	3.8233418	4.7823653	ESAM	Homo sapiens endothelial cell adhesion molecule (ESAM), mRNA [NM_138961]	NM_138961
A_23_P48088	-3.6576712	down	4.92E-02	4.927E-03	5.6825094	3.811584	5.0110226	CD27	Homo sapiens CD27 molecule (CD27), mRNA [NM_001242]	NM_001242
A_21_P0012145	3.3322854	up	2.99E-02	2.044E-03	2.121267	3.857779	2.0364542	lnc-SPTLC3-2	LNCipedia lincRNA (lnc-SPTLC3-2), lincRNA [lnc-SPTLC3-2:1]	
A_21_P0002073	2.7758617	up	4.12E-02	3.495E-03	8.650891	10.123827	9.298629		DB092709 TESTI4 Homo sapiens cDNA clone TESTI4042421 5', mRNA sequence [DB092709]	
A_33_P3404566	3.262678	up	4.47E-02	4.073E-03	4.1476	5.853657	4.204044			
A_22_P00018871	5.946908	up	1.14E-03	4.657E-06	1.9343673	4.506507	3.9860222	LINC01038	Homo sapiens long intergenic non-protein coding RNA 1038 (LINC01038), long non-coding RNA [NR_125785]	
A_33_P3215848	5.6495337	up	2.90E-02	1.942E-03	3.9538507	6.4519825	5.8248625		Homo sapiens mRNA similar to collagen, type XVII, alpha 1 (cDNA clone IMAGE:5431129). [BC044628]	BC044628
A_23_P88222	19.090704	up	4.11E-04	2.778E-07	2.7889628	7.0437613	6.891835	PLD4	Homo sapiens phospholipase D family, member 4 (PLD4), mRNA [NM_138790]	NM_138790
A_19_P00807545	2.610417	up	3.02E-02	2.072E-03	5.184612	6.568892	5.1232142		crystallin, beta B2 pseudogene 1 [Source:HGNC Symbol;Acc:HGNC:2399] [ENST00000609513]	
A_33_P3346508	9.346772	up	1.74E-03	1.695E-05	5.5102873	8.7347555	8.290354			
A_24_P461497	42.99051	up	4.39E-04	9.189E-07	4.271331	9.697277	8.856716			
A_24_P878388	2.785232	up	1.19E-02	3.680E-04	11.386931	12.864729	12.515658			
A_33_P3322125	2.1880224	up	3.44E-02	2.684E-03	3.0574234	4.187051	3.517322	DAZAP2	Homo sapiens DAZ associated protein 2 (DAZAP2), transcript variant 6, mRNA [NM_001136269]	NM_001136269
A_22_P00020157	2.5480125	up	4.89E-02	4.836E-03	4.630368	5.9797406	5.5965424	lnc-RAD1-1	Homo sapiens cDNA FLJ43326 fis, clone NT2RI3001263. [AK125316]	AK125316
A_23_P129144	10.700807	up	1.23E-02	4.065E-04	2.463688	5.8833356	5.200577	MYZAP	Homo sapiens myocardial zonula adherens protein (MYZAP), transcript variant 1, mRNA [NM_001018100]	NM_001018100
A_24_P365349	10.079133	up	3.18E-02	2.313E-03	2.0109096	5.344209	4.3731885	CACNG7	Homo sapiens calcium channel, voltage-dependent, gamma subunit 7 (CACNG7), mRNA [NM_031896]	NM_031896
A_22_P00019433	2.1770298	up	4.39E-02	3.850E-03	3.921835	5.044196	5.1350203	lnc-SLC16A3-5	LNCipedia lincRNA (lnc-SLC16A3-5), lincRNA [lnc-SLC16A3-5:1]	BX649099
A_23_P19182	3.423824	up	4.47E-02	4.003E-03	7.1975074	8.973116	9.940938	REEP2	Homo sapiens receptor accessory protein 2 (REEP2), transcript variant 2, mRNA [NM_016606]	NM_016606
A_22_P00016387	2.3858495	up	3.92E-02	3.222E-03	2.0571208	3.3116238	3.1157348			
A_24_P58242	6.0031414	up	2.47E-03	3.250E-05	4.630097	7.2158146	7.2349772		ribosomal protein S15a pseudogene 25 [Source:HGNC Symbol;Acc:HGNC:36848] [ENST00000489168]	
A_33_P3288329	13.219042	up	3.61E-02	2.869E-03	3.9246938	7.6492395	6.7581205	SORBS1	Homo sapiens sorbin and SH3 domain containing 1 (SORBS1), transcript variant 8, mRNA [NM_001290294]	NM_001290294
A_23_P29769	3.2862542	up	1.58E-02	6.303E-04	7.9976616	9.714106	9.221131	WWTR1	Homo sapiens WW domain containing transcription regulator 1 (WWTR1), transcript variant 1, mRNA [NM_015472]	NM_015472
A_22_P00025102	133.1919	up	4.39E-04	1.007E-06	1.7562649	8.813627	7.2147617			
A_33_P3355418	11.666827	up	1.12E-02	3.231E-04	2.491573	6.0359135	4.6004763	AGBL1	Homo sapiens ATP/GTP binding protein-like 1 (AGBL1), mRNA [NM_152336]	NM_152336
A_21_P0010611	5.841431	up	3.15E-02	2.250E-03	2.8043466	5.3506684	5.0627804	XLOC_l2_000900	BROAD Institute lincRNA (XLOC_l2_000900), lincRNA [TCONS_l2_00001221]	
A_23_P59099	2.9181006	up	1.62E-02	6.638E-04	4.849109	6.394139	6.3548408	OR11A1	Homo sapiens olfactory receptor, family 11, subfamily A, member 1 (OR11A1), mRNA [NM_013937]	NM_013937
A_21_P0006279	2.357354	up	1.64E-02	6.884E-04	3.9686623	5.2058306	4.539003	lnc-TRPM6-4	LNCipedia lincRNA (lnc-TRPM6-4), lincRNA [lnc-TRPM6-4:1]	
A_19_P00807614	2.2930977	up	2.73E-02	1.733E-03	3.2969725	4.4942703	3.5805511	lnc-WDR7-8	LNCipedia lincRNA (lnc-WDR7-8), lincRNA [lnc-WDR7-8:1]	
A_23_P26072	4.661514	up	7.86E-03	2.009E-04	3.4811363	5.701935	5.582512		Homo sapiens PRO1914 mRNA, complete cds. [AF118084]	AF118084
A_22_P00023095	4.1901894	up	3.98E-02	3.283E-03	2.5821211	4.6491365	4.3700504	lnc-KIF15-1	DB095685 TESTI4 Homo sapiens cDNA clone TESTI4046411 5', mRNA sequence [DB095685]	DB095685
A_22_P00020557	2.6329703	up	5.09E-03	9.956E-05	4.929719	6.3264103	6.400551	lnc-AC010536.1-1	Q9SCR0_ARATH (Q9SCR0) Scarecrow-like 7 (SCL7), partial (4%) [THC2724708]	
A_33_P3293009	3.5156019	up	2.42E-02	1.434E-03	4.7589025	6.5726743	6.1105943	NKAIN4	Homo sapiens Na+/K+ transporting ATPase interacting 4 (NKAIN4), mRNA [NM_152864]	NM_152864
A_21_P0013524	2.6647136	up	2.90E-02	1.951E-03	2.7216072	4.1355877	3.1587543	OTUD6B-AS1	PREDICTED: Homo sapiens uncharacterized LOC100506365 (GS1-251I9.4), transcript variant X3, ncRNA [XR_432334]	
A_19_P00318909	7.7862563	up	1.37E-02	5.012E-04	2.8905792	5.851509	5.3621373	XLOC_l2_010751	BROAD Institute lincRNA (XLOC_l2_010751), lincRNA [TCONS_l2_00020645]	
A_22_P00009753	2.34451	up	1.13E-02	3.276E-04	2.618812	3.8480985	3.8333719		603295654F1 NIH_MGC_96 Homo sapiens cDNA clone IMAGE:5315076 5', mRNA sequence [BI668294]	BI668294
A_22_P00010667	3.7759075	up	2.59E-02	1.614E-03	5.5164185	7.433242	7.2591505	lnc-NES-1	LNCipedia lincRNA (lnc-NES-1), lincRNA [lnc-NES-1:1]	
A_21_P0008369	26.225376	up	1.46E-02	5.663E-04	2.3623588	7.0752506	6.770934	lnc-GPR65-1	LNCipedia lincRNA (lnc-GPR65-1), lincRNA [lnc-GPR65-1:1]	
A_23_P201939	3.3036308	up	3.27E-02	2.423E-03	3.991868	5.7159204	6.4413757	PPM1J	Homo sapiens protein phosphatase, Mg2+/Mn2+ dependent, 1J (PPM1J), mRNA [NM_005167]	NM_005167
A_22_P00019426	4.7447157	up	4.58E-02	4.312E-03	3.4016252	5.647947	5.041812	lnc-RIC8A-1	LNCipedia lincRNA (lnc-RIC8A-1), lincRNA [lnc-RIC8A-1:1]	
A_19_P00317913	5.979969	up	2.47E-02	1.495E-03	2.317499	4.897637	4.884096	HLA-F-AS1	Homo sapiens HLA-F antisense RNA 1 (HLA-F-AS1), transcript variant 2, long non-coding RNA [NR_026973]	NR_026973
A_19_P00322890	2.8143983	up	4.00E-02	3.340E-03	3.4582486	4.951075	2.6780293			
A_21_P0006199	5.0319824	up	2.90E-02	1.947E-03	2.949611	5.280738	3.815021	lnc-ZNF462-1	LNCipedia lincRNA (lnc-ZNF462-1), lincRNA [lnc-ZNF462-1:3]	
A_22_P00019317	4.1526365	up	7.35E-03	1.714E-04	5.5697575	7.623785	7.377725	lnc-NMNAT3-3	LNCipedia lincRNA (lnc-NMNAT3-3), lincRNA [lnc-NMNAT3-3:2]	
A_23_P132856	39.404804	up	4.39E-04	9.815E-07	3.1768951	8.477195	8.296629	NRROS	Homo sapiens negative regulator of reactive oxygen species (NRROS), mRNA [NM_198565]	NM_198565
A_22_P00009805	8.732588	up	2.36E-02	1.365E-03	2.0221913	5.1486006	1.791163			
A_21_P0004989	6.6942263	up	2.07E-02	1.018E-03	2.7766778	5.519595	4.259922	lnc-GMDS-1	LNCipedia lincRNA (lnc-GMDS-1), lincRNA [lnc-GMDS-1:1]	
A_24_P92267	117.67072	up	7.79E-04	2.185E-06	2.870093	9.748705	9.02667			
A_33_P3213685	2.2339394	up	4.92E-02	4.901E-03	2.1939337	3.3535237	1.8346009	CAB39L	Homo sapiens calcium binding protein 39-like (CAB39L), transcript variant 4, mRNA [NM_001287339]	NM_001287339
A_23_P23443	2.3167999	up	3.45E-02	2.701E-03	10.754271	11.966404	11.655912	EFHD2	Homo sapiens EF-hand domain family, member D2 (EFHD2), mRNA [NM_024329]	NM_024329
A_24_P772488	5.9351215	up	3.35E-02	2.537E-03	2.7316477	5.3009253	4.6594167	PLXNA4	Homo sapiens plexin A4 (PLXNA4), transcript variant 1, mRNA [NM_020911]	NM_020911
A_22_P00020378	2.2046416	up	3.18E-02	2.292E-03	2.412814	3.553358	3.171226		Homo sapiens fusion RNA for switch circle transcript (Igamma2 - Cmu) in t(11;14) mantle cell lymphoma. [AJ617578]	AJ617578
A_32_P466514	2.077722	up	7.10E-03	1.628E-04	5.8978157	6.9528184	6.5298705	IRF2BPL	Homo sapiens interferon regulatory factor 2 binding protein-like (IRF2BPL), mRNA [NM_024496]	NM_024496
A_22_P00000718	3.484222	up	2.36E-02	1.379E-03	2.2457209	4.0465574	4.1775093	lnc-ADAMTS18-1	LNCipedia lincRNA (lnc-ADAMTS18-1), lincRNA [lnc-ADAMTS18-1:1]	
A_21_P0014129	4.7097425	up	4.07E-02	3.438E-03	2.4652128	4.700861	3.6792235	LINC00578	Homo sapiens long intergenic non-protein coding RNA 578 (LINC00578), long non-coding RNA [NR_047568]	NR_047568

A_23_P383258	7.671959	up	2.02E-02	9.797E-04	1.7330625	4.6726575	2.8554714	GDA	Homo sapiens guanine deaminase (GDA), transcript variant 2, mRNA [NM_004293]	NM_004293
A_23_P368740	3.6407819	up	4.92E-02	4.946E-03	6.1546097	8.018858	6.80281	HDAC10	Homo sapiens histone deacetylase 10 (HDAC10), transcript variant 1, mRNA [NM_032019]	NM_032019
A_23_P123164	5.783192	up	1.78E-02	7.961E-04	3.1745796	5.7064457	5.047138	OR6W1P	Homo sapiens olfactory receptor, family 6, subfamily W, member 1 pseudogene (OR6W1P), non-coding RNA [NR_002140]	NR_002140
A_23_P257649	11.458492	up	2.32E-02	1.298E-03	5.320437	8.838782	7.8562336	RBP1	Homo sapiens retinol binding protein 1, cellular (RBP1), transcript variant 1, mRNA [NM_002899]	NM_002899
A_33_P3253892	2.1581197	up	3.09E-02	2.145E-03	1.8804418	2.9902167	2.8358524			
A_23_P7048	10.464686	up	4.30E-02	3.719E-03	2.2061675	5.5936246	4.447854	LINC01587	Homo sapiens long intergenic non-protein coding RNA 1587 (LINC01587), transcript variant 2, long non-coding RNA [NR_126518]	NR_126518
A_33_P3257784	4.876127	up	2.90E-02	1.915E-03	5.2292395	7.514975	6.950926	TSPAN17	Homo sapiens tetraspanin 17 (TSPAN17), transcript variant 1, mRNA [NM_012171]	NM_012171
A_22_P00004895	3.8380644	up	3.15E-02	2.261E-03	2.3484523	4.288831	3.081831			
A_24_P228717	5.4685345	up	3.75E-03	6.024E-05	5.7471204	8.198275	8.136852	RAC2	Homo sapiens ras-related C3 botulinum toxin substrate 2 (rho family, small GTP binding protein Rac2) (RAC2), mRNA [NM_002872]	NM_002872
A_21_P0009305	3.065369	up	3.59E-03	5.477E-05	5.0658555	6.681916	6.403196	lnc-MFAP4-4	LNCipedia lincRNA (lnc-MFAP4-4), lincRNA [lnc-MFAP4-4:1]	
A_23_P412476	5.078423	up	2.86E-02	1.870E-03	2.537288	4.8816686	5.087158	GREB1	Homo sapiens growth regulation by estrogen in breast cancer 1 (GREB1), transcript variant c, mRNA [NM_148903]	NM_148903
A_22_P00012461	2.7294452	up	1.65E-02	7.101E-04	2.3804312	3.8290389	3.6655369	lnc-PRR14-1	LNCipedia lincRNA (lnc-PRR14-1), lincRNA [lnc-PRR14-1:1]	
A_32_P494620	12.494072	up	3.88E-03	6.434E-05	2.236792	5.879964	5.4176073	LHFPL5	Homo sapiens lipoma HMGIC fusion partner-like 5 (LHFPL5), mRNA [NM_182548]	NM_182548
A_33_P3420416	3.3692245	up	1.37E-02	5.021E-04	5.2378306	6.9902472	6.562834	LGALS9	Homo sapiens lectin, galactoside-binding, soluble, 9 (LGALS9), transcript variant 2, mRNA [NM_002308]	NM_002308
A_21_P0000710	7.7853985	up	1.74E-03	1.695E-05	2.0931165	5.0538874	5.4320097	HMGA1P7	Homo sapiens high mobility group AT-hook 1 pseudogene 7 (HMGA1P7), non-coding RNA [NR_037938]	NR_037938
A_24_P74559	16.083923	up	4.66E-02	4.397E-03	3.0770853	7.0846324	6.3748426	CYTH4	Homo sapiens cytohesin 4 (CYTH4), mRNA [NM_013385]	NM_013385
A_22_P00018081	2.7510715	up	2.31E-02	1.223E-03	3.2176487	4.6776423	4.3462353	LOC101929464	Homo sapiens uncharacterized LOC101929464 (LOC101929464), long non-coding RNA [NR_125978]	NR_125978
A_24_P940125	3.0624576	up	3.13E-02	2.195E-03	7.3938694	9.008559	8.831198	CNOT6	Homo sapiens CCR4-NOT transcription complex, subunit 6 (CNOT6), mRNA [NM_001303241]	NM_001303241
A_21_P0008066	2.2563584	up	1.36E-02	4.920E-04	3.0515296	4.225526	3.0211074	lnc-OLFM4-2	LNCipedia lincRNA (lnc-OLFM4-2), lincRNA [lnc-OLFM4-2:1]	
A_21_P0000492	2.533174	up	3.18E-02	2.318E-03	11.481149	12.822095	11.65675	SNAR-F	Homo sapiens small ILF3/NF90-associated RNA F (SNAR-F), small nuclear RNA [NR_004384]	NR_004384
A_21_P0001512	2.0807045	up	2.47E-03	3.125E-05	1.8863164	2.9433885	3.8345582	LOC101930559	PREDICTED: Homo sapiens uncharacterized LOC101930559 (LOC101930559), ncRNA [XR_425171]	
A_21_P0007803	8.884324	up	1.45E-02	5.443E-04	2.4232943	5.5745564	5.3131495	lnc-TMEM132D-2	LNCipedia lincRNA (lnc-TMEM132D-2), lincRNA [lnc-TMEM132D-2:1]	
A_33_P3336462	2.4863243	up	4.92E-02	4.897E-03	4.799469	6.1134834	4.878437		Homo sapiens cDNA FLJ41306 fis, clone BRAMY2042549. [AK123300]	AK123300
A_33_P3286616	2.3660972	up	4.70E-02	4.460E-03	6.759497	8.002007	7.613921	IRAK1	Homo sapiens interleukin-1 receptor-associated kinase 1 (IRAK1), transcript variant 1, mRNA [NM_001569]	NM_001569
A_23_P45338	3.3386595	up	1.33E-02	4.717E-04	1.9574516	3.6967206	3.1297889	EFHC2	Homo sapiens EF-hand domain (C-terminal) containing 2 (EFHC2), mRNA [NM_025184]	NM_025184
A_22_P00016489	2.3329341	up	7.49E-03	1.777E-04	4.3653407	5.5874863	5.1740303	LOC102724715	PREDICTED: Homo sapiens uncharacterized LOC102724715 (LOC102724715), ncRNA [XR_424811]	XR_424811
A_33_P3383861	4.655555	up	1.62E-02	6.615E-04	2.63656	4.855513	4.7666225	LOC646471	Homo sapiens uncharacterized LOC646471 (LOC646471), long non-coding RNA [NR_024498]	NR_024498
A_33_P3258349	2.281593	up	4.66E-03	8.672E-05	1.9420912	3.1321328	2.2731915	LOC644145	Homo sapiens exocyst complex component 1 pseudogene (LOC644145), non-coding RNA [NR_003935]	NR_003935
A_21_P0010095	146.7572	up	4.39E-04	6.575E-07	1.877927	9.075214	7.6623445	lnc-CEBPB-6	LNCipedia lincRNA (lnc-CEBPB-6), lincRNA [lnc-CEBPB-6:1]	
A_33_P3393801	15.849773	up	7.86E-03	2.025E-04	3.112498	7.0988884	6.622931	PDZK1IP1	Homo sapiens PDZK1 interacting protein 1 (PDZK1IP1), mRNA [NM_005764]	NM_005764
A_23_P202071	15.37133	up	2.84E-02	1.847E-03	4.5045166	8.446687	7.8114076	CELF2	Homo sapiens CUGBP, Elav-like family member 2 (CELF2), transcript variant 3, mRNA [NM_001025077]	NM_001025077
A_33_P3405429	2.455445	up	2.02E-02	9.909E-04	2.5630693	3.8590539	3.1144032	VWA5B1	von Willebrand factor A domain containing 5B1 [Source:HGNC Symbol;Acc:HGNC:26538] [ENST00000473325]	AK125833
A_19_P00805452	3.5441072	up	4.85E-02	4.778E-03	4.325754	6.1511765	4.6590147	XLOC_l2_010558	BROAD Institute lincRNA (XLOC_l2_010558), lincRNA [TCONS_l2_00020391]	
A_21_P0010013	4.759893	up	4.41E-02	3.924E-03	2.3715541	4.6224833	3.5313966	lnc-FOXA2-5	LNCipedia lincRNA (lnc-FOXA2-5), lincRNA [lnc-FOXA2-5:1]	
A_33_P3316953	6.369514	up	2.51E-02	1.534E-03	1.7852294	4.456413	4.0258746	RBFOX3	Homo sapiens RNA binding protein, fox-1 homolog (C. elegans) 3 (RBFOX3), mRNA [NM_001082575]	NM_001082575
A_33_P3279820	3.2992637	up	1.72E-02	7.581E-04	4.1282396	5.8503838	6.023707			
A_24_P409519	2.018356	up	2.31E-02	1.212E-03	1.7871389	2.8003197	2.5319307	PTPN5	Homo sapiens protein tyrosine phosphatase, non-receptor type 5 (striatum-enriched) (PTPN5), transcript variant 1, mRNA [NM_006906]	NM_006906
A_22_P00004509	8.620757	up	1.74E-03	1.392E-05	2.1673658	5.2751803	4.655482	lnc-CPT2-4	LNCipedia lincRNA (lnc-CPT2-4), lincRNA [lnc-CPT2-4:1]	
A_22_P00005942	5.906219	up	4.30E-02	3.705E-03	4.0602	6.622435	6.126542	lnc-EXOC3L4-1	CR736179 Soares_fetal_lung_NbHL19W Homo sapiens cDNA clone IMAGp971A1684 ; IMAGE:1952157 5', mRNA sequence [CR736179]	CR736179
A_22_P00005443	18.053059	up	5.82E-03	1.216E-04	2.5595188	6.7336903	6.4018784	lnc-DUSP1-2	DB065448 TESTI4 Homo sapiens cDNA clone TESTI4006501 5', mRNA sequence [DB065448]	DB065448
A_33_P3380751	2.4584491	up	1.46E-02	5.650E-04	5.7875023	7.085251	6.8084297	ST8SIA1	Homo sapiens ST8 alpha-N-acetyl-neuraminide alpha-2,8-sialyltransferase 1 (ST8SIA1), mRNA [NM_003034]	NM_003034
A_23_P87310	15.263365	up	1.00E-02	2.831E-04	2.6774354	6.6094365	5.9871864	LMO1	Homo sapiens LIM domain only 1 (rhombotin 1) (LMO1), transcript variant 1, mRNA [NM_002315]	NM_002315
A_21_P0014220	2.091639	up	3.80E-02	3.103E-03	2.0982833	3.1629171	1.8546379		carbonic anhydrase XIII [Source:HGNC Symbol;Acc:HGNC:14914] [ENST00000518392]	XR_108904
A_21_P0013342	2.818014	up	2.84E-02	1.839E-03	2.2815654	3.7762442	4.06146	CCZ1	Homo sapiens CCZ1 vacuolar protein trafficking and biogenesis associated homolog (S. cerevisiae) (CCZ1), mRNA [NM_015622]	NM_015622
A_22_P00007142	3.3187554	up	3.32E-02	2.485E-03	10.519051	12.249693	11.919773		Q8LCA6_ARATH (Q8LCA6) Transcriptional coactivator-like protein, partial (7%) [THC2683788]	BC033528
A_33_P3343577	2.414751	up	4.84E-02	4.718E-03	1.9467041	3.2185786	2.7416		T cell receptor beta variable 10-3 [Source:HGNC Symbol;Acc:HGNC:12179] [ENST00000611462]	AB305952
A_33_P3354940	3.3066874	up	4.84E-02	4.690E-03	5.0031548	6.7285414	6.4113398	CSF1	Homo sapiens colony stimulating factor 1 (macrophage) (CSF1), transcript variant 1, mRNA [NM_000757]	NM_000757
A_21_P0013026	5.5603094	up	4.39E-04	9.863E-07	1.9420512	4.4172163	4.0169835	XLOC_l2_012578	BROAD Institute lincRNA (XLOC_l2_012578), lincRNA [TCONS_l2_00024254]	
A_21_P0007452	4.010085	up	2.67E-02	1.686E-03	3.4647996	5.4684324	5.2063265	lnc-AP000679.2.1-1	LNCipedia lincRNA (lnc-AP000679.2.1-1), lincRNA [lnc-AP000679.2.1-1:1]	
A_32_P25514	4.6331387	up	2.39E-03	2.905E-05	3.6643367	5.8763266	5.79671	GABRG2	Homo sapiens gamma-aminobutyric acid (GABA) A receptor, gamma 2 (GABRG2), transcript variant 1, mRNA [NM_198904]	NM_198904
A_21_P0009890	3.3435168	up	1.45E-02	5.391E-04	4.635953	6.3773193	6.0573063			

A_23_P141035	2.9563332	up	4.47E-02	4.014E-03	5.151186	6.714995	5.750703	CHST4	Homo sapiens carbohydrate (N-acetylglucosamine 6-O) sulfotransferase 4 (CHST4), transcript variant 1, mRNA [NM_005769]	NM_005769
A_21_P0014280	7.5137196	up	2.19E-02	1.113E-03	2.914421	5.8239484	6.084383	LOC100128276	Homo sapiens cDNA FLJ43282 fis, clone LIVER2007415. [AK125272]	AK125272
A_23_P353614	2.9199715	up	4.41E-02	3.907E-03	4.287288	5.8332424	6.814702	C8orf46	Homo sapiens chromosome 8 open reading frame 46 (C8orf46), mRNA [NM_152765]	NM_152765
A_24_P136094	2.6014712	up	4.27E-02	3.669E-03	5.734892	7.1142197	6.4612913	CCDC88C	Homo sapiens coiled-coil domain containing 88C (CCDC88C), mRNA [NM_001080414]	NM_001080414

Supplementary Table 4. Most relevant networks involving the genes that are deregulated in PDX *vs.* primary OS tumors

Network name	Processes	Size	Target	Pathways	p-Value	zScore	gScore
**PU.1, MANR, CD163, C1qRp, HLA-DQB1**	immune response (83.7%), defense response (79.6%), immune system process (89.8%), regulation of immune system process (77.6%), positive regulation of immune system process (69.4%)	50	25	1	8.38E-56	83.72	84.97
**VEGFR-2, VEGFR-1, COX-1 (PTGS1), CSF2RA, NF-AT1 (NFATC2)**	response to oxygen-containing compound (86.0%), intracellular signal transduction (81.4%), positive regulation of protein phosphorylation (69.8%), response to organic substance (95.3%), positive regulation of phosphorylation (69.8%)	59	4	48	2.47E-06	12.88	72.88
**c-Jun, H2-Ab-1**	antigen processing and presentation of peptide or polysaccharide antigen via MHC class II (89.6%), antigen processing and presentation of exogenous peptide antigen via MHC class II (83.3%), antigen processing and presentation of peptide antigen via MHC class II (83.3%), antigen processing and presentation (89.6%), antigen processing and presentation of exogenous peptide antigen (83.3%)	50	13	0	1.40E-29	60.36	60.36

Supplementary Table 5. Enrichment by Pathway Maps.

Maps	In Data	Total	p-value	FDR	Network Objects from Active Data
Immune response_Antigen presentation by MHC class I: cross-presentation	14	99	1.15731E-10	5.48564E-08	HSP70, TLR7, VAMP8, VAV-1, MANR, gp91-phox, SREC-I, Fc epsilon RI gamma, HSPA1A, Fc gamma RII alpha, p47-phox, Rac2, DAP12, CD74
Inhibition of neutrophil migration by proresolving lipid mediators in COPD	11	71	4.5857E-09	1.08681E-06	p38 MAPK, alpha-M/beta-2 integrin, VAV-1, PECAM1, CD34, PI3K cat class IB (p110-gamma), Rac2, FPR, PTAFR, PLC-beta2, ITGB2
Cell adhesion_Integrin inside-out signaling in neutrophils	11	77	1.11167E-08	1.75644E-06	p38 MAPK, alpha-M/beta-2 integrin, PI3K cat class IB (p110-gamma), PREL1, Slp76, DAP12, FPR, PTAFR, PLC-beta2, ITGB2, SLAP-130(ADAP)
Immune response_Antigen presentation by MHC class II	11	118	9.62545E-07	0.000114062	MHC class II, MHC class II alpha chain, p38 MAPK, HLA-DM, PSD4, HCLS1, Fc gamma RII beta, MHC class II beta chain, MANR, Fc epsilon RI gamma, CD74
Oxidative stress_Activation of NADPH oxidase	8	59	1.81236E-06	0.000154941	p38 MAPK, VAV-1, gp91-phox, PI3K cat class IB (p110-gamma), p40-phox, p47-phox, Rac2, PLC-beta
Immune response_PIP3 signaling in B lymphocytes	7	42	1.96128E-06	0.000154941	DAPP1, VAV-1, Fc gamma RII beta, PI3K cat class IB (p110-gamma), CD45, PLC-beta, p90Rsk
Role of cell adhesion in vaso-occlusion in Sickle cell disease	7	43	2.31465E-06	0.000156735	alpha-M/beta-2 integrin, ITGAM, PECAM1, CD14, Fc gamma RII alpha, CD45, ITGB2
Oxidative stress_Role of IL-8 signaling pathway in respiratory burst	7	48	4.98048E-06	0.000295093	p38 MAPK, gp91-phox, PI3K cat class IB (p110-gamma), p40-phox, p47-phox, Rac2, PLC-beta2
Rheumatoid arthritis (general schema)	7	50	6.59615E-06	0.000347397	MHC class II, CD2, HLA-DRB1, HLA-DRB, MHC class II beta chain, HLA-DRB4, CSF1
Immune response_IL-5 signaling via PI3K, MAPK and NF-kB	8	76	1.25429E-05	0.000594532	p38 MAPK, alpha-M/beta-2 integrin, ITGAM, Fc gamma RII beta, Fc gamma RII alpha, p47-phox, PTAFR, ITGB2

Supplementary Figure Legends

Supplementary Figure 1.

Comparison of PDX growth in two immunodeficient mouse strains. The same *in vivo* passage of six OS and three EW was implanted in one NSG and one RGKO mouse. Each bar represents the mean tumor latency in weeks ± SEM. n.s., not significant by the Student's paired *t* test (p=0.371 for OS and p=0.326 for EW).

Supplementary Figure 2.

Unsupervised Principal Component Analysis of OS (blue) and EW (red) including clinical samples (squares), PDX (circles) and *in vitro* cultures (triangles). Parameters: column indices= [1-20], pruning option= [numPrincipalComponents, [4]], mean centered=true, scale=true, 3D scores=true, PCA on=Columns.

Supplementary Figure 1

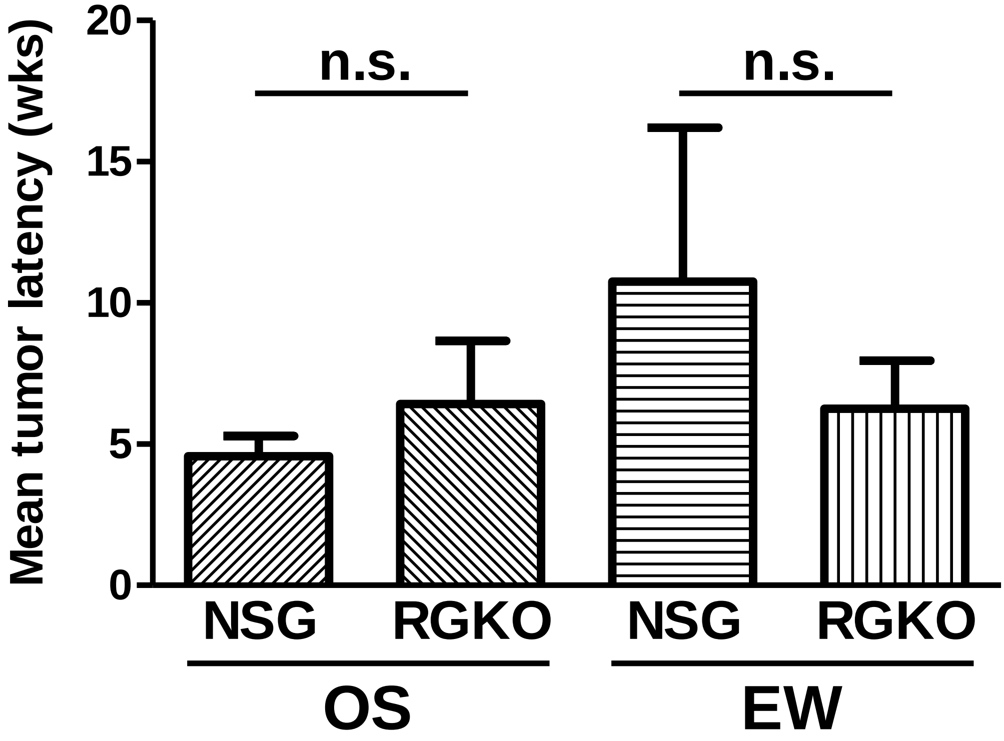

Supplementary Figure 2

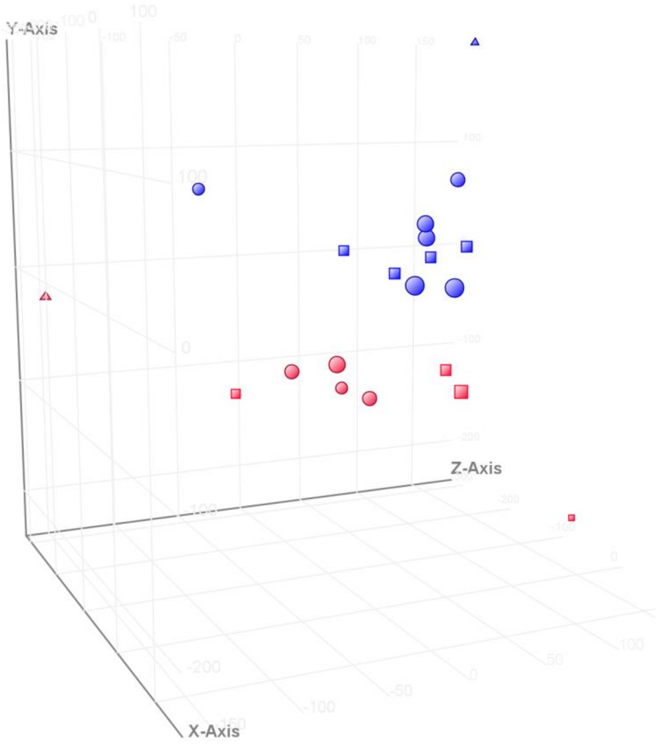

Supplement: Supplementary file 1 — Supplementary files [file 41598_2019_48634_MOESM1_ESM.pdf]
